# Eugenol@natural Zeolite vs. Citral@natural Zeolite Nanohybrids for Gelatin-Based Edible-Active Packaging Films

**DOI:** 10.3390/gels11070518

**Published:** 2025-07-03

**Authors:** Achilleas Kechagias, Areti A. Leontiou, Yelyzaveta K. Oliinychenko, Alexandros Ch. Stratakos, Konstantinos Zaharioudakis, Katerina Katerinopoulou, Maria Baikousi, Nikolaos D. Andritsos, Charalampos Proestos, Nikolaos Chalmpes, Aris E. Giannakas, Constantinos E. Salmas

**Affiliations:** 1 Department of Food Science and Technology, University of Patras, 30100 Agrinio, Greece; up1110842@upatras.gr (A.K.); aleontiu@upatras.gr (A.A.L.); zacharioudakis.k@upatras.gr (K.Z.); akaterin@upatras.gr (K.K.); nandritsos@upatras.gr (N.D.A.); 2 School of Applied Sciences, College for Health, Science and Society, University of the West of England, Coldharbour Ln, Bristol BS16 1QY, UK; yelyzaveta2.oliinychenko@live.uwe.ac.uk (Y.K.O.); alexandros.stratakos@uwe.ac.uk (A.C.S.); 3 Department of Material Science and Engineering, University of Ioannina, 45110 Ioannina, Greece; mbaikou@uoi.gr; 4 Laboratory of Food Chemistry, Department of Chemistry, National and Kapodistrian University of Athens Zografou, 15771 Athens, Greece; harpro@chem.uoa.gr; 5 Department of Materials Science and Engineering, Cornell University, Ithaca, NY 14850, USA

**Keywords:** gelatin, eugenol, citral, natural zeolite, edible packaging, active packaging, zero oxygen barrier, pork ham, shelf life

## Abstract

In this study, aligned with the principles of the circular economy and sustainability, novel eugenol@natural zeolite (EG@NZ) and citral@natural zeolite (CT@NZ) nanohybrids were developed. These nanohybrids were successfully incorporated into a pork gelatin (Gel)/glycerol (Gl) composite matrix using an extrusion–compression molding method to produce innovative active packaging films: Gel/Gl/xEG@NZ (where x = 5, 10, and 15%wt.) and Gel/Gl/xCT@NZ (where x = 5 and 10%wt.). All films exhibited zero oxygen barrier properties. Release kinetic studies showed that both EG@NZ and CT@NZ nanohybrids adsorbed up to 58%wt. of their respective active compounds. However, EG@NZ exhibited a slow and nearly complete release of eugenol, whereas CT@NZ released approximately half of its citral content at a faster rate. Consequently, the obtained Gel/Gl/xEG@NZ films demonstrated significantly higher antioxidant activity as measured by the 2,2-diphenyl-1-picrylhydrazylradical (DPPH) assay and superior antibacterial effectiveness against *Escherichia coli* and *Listeria monocytogenes* compared to their CT-based counterparts. Overall, the Gel/Gl/xEG@NZ films show strong potential for applications as active pads for fresh pork ham slices, offering zero oxygen permeability, enhanced antioxidant and antibacterial properties, and effective control of total viable count (TVC) growth, maintaining a low and steady rate beyond the 10th day of a 26-day storage period.

## 1. Introduction

Food packaging is a field that consistently attracts both scientific and industrial interest due to its critical role in protecting food quality and ensuring effective communication with consumers [1]. Specifically, it plays a vital role in ensuring compliance with regulatory standards and consumer expectations regarding food safety, nutritional value, and organoleptic properties. As such, researchers continue to focus on meeting these requirements while keeping pace with modern trends in the sector [2,3,4].

To meet these objectives, it is essential to address current challenges facing both the food industry and society. Chief among these is the reliance on non-biodegradable plastics in food packaging [5], which contributes significantly to environmental pollution. Over the past 70 years, approximately 8.3 billion tons of plastic have been produced [3], much of which ends up in ecosystems where it can persist for centuries, posing risks to wildlife and human health. This issue is compounded by the fact that conventional plastics are petroleum-based, relying on non-renewable resources [2]. Another major concern is the impact on human health from hazardous food and packaging additives. Synthetic chemical preservatives have been linked to serious health conditions, such as cancer, obesity, cardiovascular disease, and asthma. Furthermore, harmful substances can migrate from packaging materials into food [6,7]. In parallel, the industry faces pressure to extend the shelf life of food products to address the increasing global demand and reduce significant food waste that occurs before products even reach consumers [2,8].

To confront these difficulties, researchers are prioritizing the development of innovative, sustainable packaging solutions that promote both human and environmental health. The primary focus is on sustainable food packaging made from novel, biodegradable materials derived from renewable sources such as food waste and biomass [2,9]. These materials decompose through microbial action and can serve as a foundation for active packaging systems that reduce or eliminate the need for synthetic preservatives by incorporating natural, bio-based compounds with antimicrobial and antioxidant functions [9,10,11].

One biopolymer that has garnered considerable attention for food packaging is gelatin. Gelatin (Gel) is a natural polymer obtained from the hydrolytic breakdown of collagen under acidic (type A) or alkaline (type B) conditions [12]. It is derived from animal cartilage, bones, and skin [2] and is known for its biodegradability, low cost, and film-forming ability. Gel also provides good oxygen barrier properties and is widely used in the food industry for its emulsifying and stabilizing functions [13,14]. Researchers have enhanced Gel films by incorporating natural compounds and nanocomposites, resulting in improved mechanical, antimicrobial, and antioxidant properties and extended shelf life of packaged food products [15,16,17,18]. While casting is the traditional method for Gel film preparation, extrusion molding offers a faster, more scalable alternative suitable for industrial applications [18,19,20,21,22].

However, Gel films are inherently brittle, necessitating the use of plasticizers to improve their flexibility and mechanical properties. Plasticizers work by inserting themselves between polymer chains, reducing intermolecular forces and increasing elasticity and stretchability [23,24,25]. Glycerol (Gl), a bio-based, biodegradable, and edible plasticizer, has been successfully used in gelatin films [26,27]. Its hydrophilic nature allows it to integrate with gelatin macromolecules, increasing flexibility [23]. Gl is an environmentally friendly, low-cost byproduct of biodiesel production [28].

To further enhance packaging films with active compounds, a nanocarrier is required to ensure uniform distribution within the polymer matrix and controlled release [29]. Zeolites are crystalline aluminosilicates with a porous structure and can be either natural or synthetic [11,29,30,31]. Natural zeolite (NZ) serves as an efficient carrier material owing to its distinctive structural and chemical characteristics, which allow it to interact with a diverse array of substances, thereby rendering it beneficial for numerous applications [32,33]. In particular, its specific surface area, biocompatibility, edibility, adsorption capacity, cation-exchange capacity, porosity, and thermal stability position it as an outstanding carrier for volatile compounds like essential oils (EOs) [34]. NZ has recently been used successfully as a carrier for EOs, such as thymol [15] and carvacrol [11] in packaging materials, resulting in extended shelf life of pork products.

EOs are promising natural additives for food packaging due to their low toxicity and preservative properties [10,35,36]. Eugenol (EG), a phenylpropanoid and the main constituent of clove oil, is notable for its antimicrobial and antioxidant activity. It is already used in the pharmaceutical industry and as a flavoring agent in food and beverages [37,38]. Citral (CT), another highly promising EO found in lemongrass and various citrus fruits, exists as two isomers, geranial (trans-citral) and neral (cis-citral), and is also known for its antimicrobial effects [39,40]. Both EG and CT have demonstrated effectiveness in inhibiting common foodborne pathogens such as *Listeria monocytogenes* and *Escherichia coli* [41].

These pathogens are among the most concerning foodborne microorganisms causing serious illness in humans and representing major public health challenges [42,43]. Despite technological advancements, the incidence of foodborne diseases has risen over the past two decades, largely due to intensified food production and global population growth, which have complicated supply chains and increased contamination risks [44,45]. Thus, the development of antimicrobial biodegradable films presents a promising strategy to enhance food safety during storage.

In this study, we report the development of type A Gel/Gl composite films, enhanced with novel nanohybrids of natural zeolite loaded with either eugenol (EG@NZ) or citral (CT@NZ). Both the nanohybrids and the resulting films were thoroughly characterized and compared. Specifically, we present for the first time: (i) the synthesis and characterization of EG@NZ and CT@NZ nanohybrids, including release kinetics and physicochemical analysis using X-ray diffraction (XRD), Fourier-transform infrared spectroscopy (FTIR), and scanning electron microscopy (SEM), and (ii) the development of Gel/Gl/xEG@NZ (x = 5, 10, and 15 wt.%) and Gel/Gl/xCT@NZ (x = 5 and 10 wt.%) active packaging films via extrusion–compression molding, along with comprehensive evaluation of their structure (XRD, FTIR, SEM), oxygen barrier properties, antioxidant activity (via DPPH assay), and antibacterial performance against *E. coli* and *L. monocytogenes*.

## 2. Results and Discussion

### 2.1. EG and CT Release Kinetics

**Figure 1:**
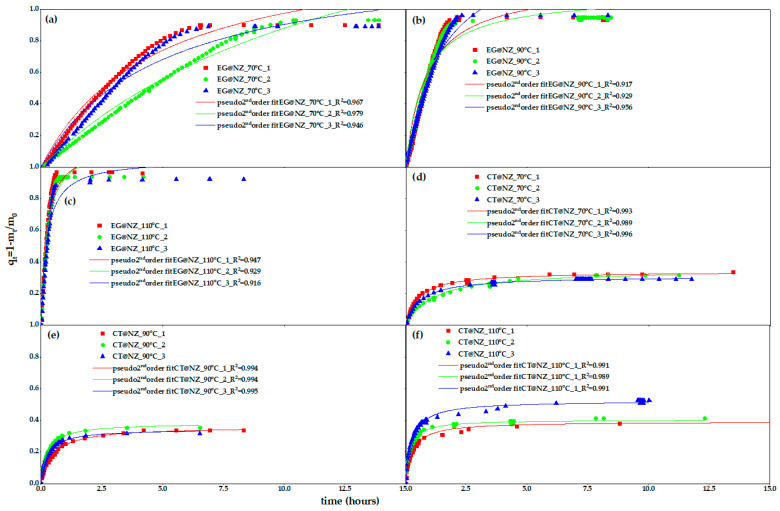
Desorption isotherm kinetic plots of EG and CT (in triplicate) for EG@NZ (**left** panels: plots **a**–**c**) and CT@NZ (**right** panels: plots **d**–**f**) nanohybrids at 70 °C (**a**,**d**), 90 °C (**b**,**e**), and 110 °C (**c**,**f**). Solid lines represent model fits based on the pseudo-second-order kinetic model.

Figure 1 shows the recorded values of (q_t_ = 1 − m_t_/m_0_) as a function of time (t) measured in triplicate at 70, 90, and 110 °C for both EG@NZ and CT@NZ nanohybrids. The dependent dimensionless variable *qₜ* represents the fraction of EOs desorbed after time *t*, relative to the total adsorbed amount *m*_0_. Similarly, the dimensionless parameter *qₑ* denotes the final overall fraction of EOs desorbed at the end of the desorption process, also relative to *m*_0_.

The experimental data were fitted using the pseudo-order kinetic model, as described in the Supplementary Materials. As shown by the R^2^ values presented in Figure S1, the pseudo-second-order kinetic model provided a good fit in all cases. The rate constant *k*_2_ (s^−1^) of the pseudo-second-order model and the equilibrium desorption capacity *qₑ* were calculated from the fitting and are summarized in Table 1 for comparison. The desorption rate constant *k*_2_ (s^−1^) represents the desorption rate of EG and CT.

As shown during the preparation of the EG@NZ and CT@NZ nanohybrids, both EG and CT were loaded onto the NZ matrix at approximately 58%wt, which is expected given the similar molecular sizes of EG and CT and their adsorption onto the NZ framework. According to the results in Table 1, the EG@NZ nanohybrid desorbed approximately 90, 93, and 95% of the adsorbed EG at 70, 90, and 110 °C respectively. In contrast, the CT@NZ nanohybrid released only about 33, 36, and 44% of the adsorbed CT under the same temperature conditions. In other words, EG@NZ releases nearly all the adsorbed EG, while CT@NZ desorbs less than half of the adsorbed CT. This suggests that a fraction of the CT molecules is strongly bound to the NZ surface, likely due to hydrogen bonding between the aldehyde group of CT and the hydroxyl groups of NZs.

**Figure 2:**
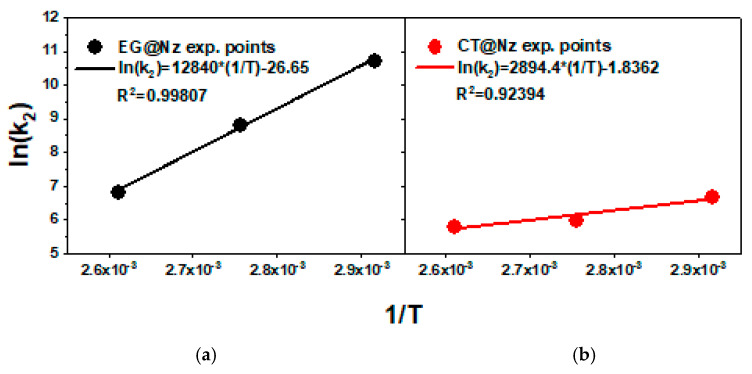
ln(1/k_2_) values as a function of (1/T) plots for (**a**) EG@NZ and (**b**) CT@NZ nanohybrids.

Based on the calculated k_2_ values, the ln(1/k_2_) was plotted as a function of 1/T for both EG@NZ and CT@NZ nanohybrids, as shown in Figure 2. From the linear fits shown in Figure 2 and the corresponding linear equations, the calculated slopes were used in conjunction with Equations (S2) and (S3) provided in the Supplementary Materials to determine the desorption energies (E_0_,_des_) of EG and CT. The estimated values were 25.2 kcal/mol for EG@NZ and 5.6 kcal/mol for CT@NZ. These results indicate that EG molecules exhibit stronger interactions with the NZ matrix compared to CT molecules.

### 2.2. XRD Analysis

**Figure 3:**
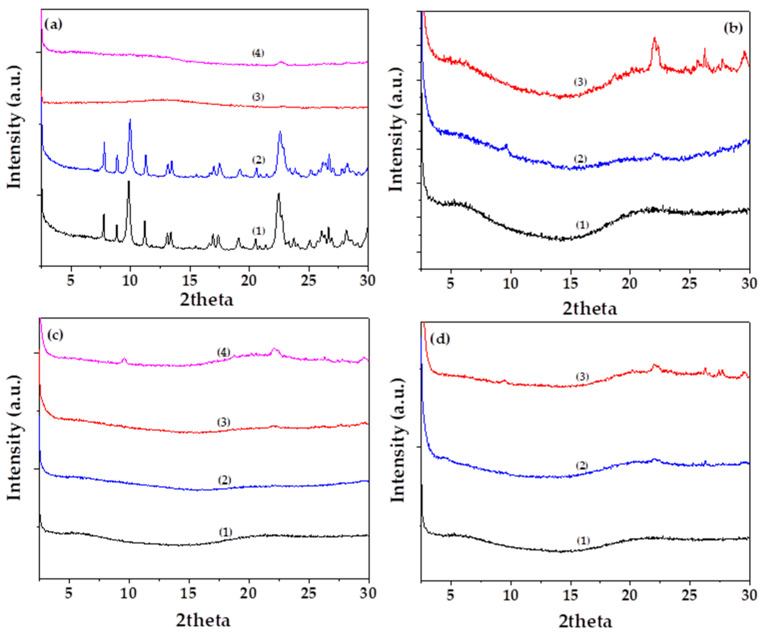
(**a**) XRD diffractograms of (1) raw natural zeolite (NZ), (2) vacuum-dried NZ, (3) EG@NZ nanohybrid, and (4) CT@NZ nanohybrid, (**b**) XRD diffractograms of (1) Gel/Gl25, (2) Gel/Gl25/NZ5, and (3) Gel/Gl25/NZ10, (**c**) XRD diffractograms of (1) Gel/Gl25, (2) Gel/Gl25/EG@NZ5, (3) Gel/Gl25/EG@NZ10, and (4) Gel/Gl25/EG@NZ15, (**d**) XRD diffractograms of (1) Gel/Gl25, (2) Gel/Gl25/CT@NZ5, and (3) Gel/Gl25/CT@NZ10.

Figure 3a presents the XRD patterns of (1) as-received natural zeolite (NZ), (2) vacuum-dried NZ, and the nanohybrids (3) EG@NZ and (4) CT@NZ, shown for comparison. As observed in the XRD pattern of pure NZ, the characteristic reflections corresponding to the heulandite Ca(Si_7_Al_2_)O_16_·6H_2_O monoclinic crystal phase (PDF-41-1357) [11,46] are observed. As expected, no changes in the crystalline phase of NZ are detected after vacuum drying, indicating that the drying process does not alter the structural integrity of the zeolite.

In contrast, the XRD patterns of both EG@NZ and CT@NZ nanohybrids show a near-complete disappearance of the characteristic heulandite reflections. This suggests that the NZ structure becomes saturated and coated with the EOs, such that its crystalline framework is no longer detectable via XRD analysis.

Figure 3b–d present the XRD patterns of Gel/Gl/xNZ, Gel/Gl/xEG@NZ, and Gel/Gl/xCT@NZ films, respectively, for comparison. All films exhibit predominantly amorphous profiles. Only in the case of Gel/Gl/10NZ does the reflection associated with NZ appear clearly, indicating limited dispersion. The absence of detectable NZ reflections in the EG@NZ- and CT@NZ-containing films suggests that modification of NZ with EG and CT significantly enhances the dispersion of the nanohybrids within the Gel/Gl matrix. This observation is in line with previous studies [15,47,48]. Additionally, the fact that the addition of both EG@NZ and CT@NZ nanohybrids does not affect the amorphous phase of the Gel/Gl matrix implies that no significant interactions occur between the nanohybrids and the Gel/Gl matrix that could potentially alter the amorphous phase [49,50].

### 2.3. Fourier-Transform Infrared (FTIR) Spectroscopy

**Figure 4:**
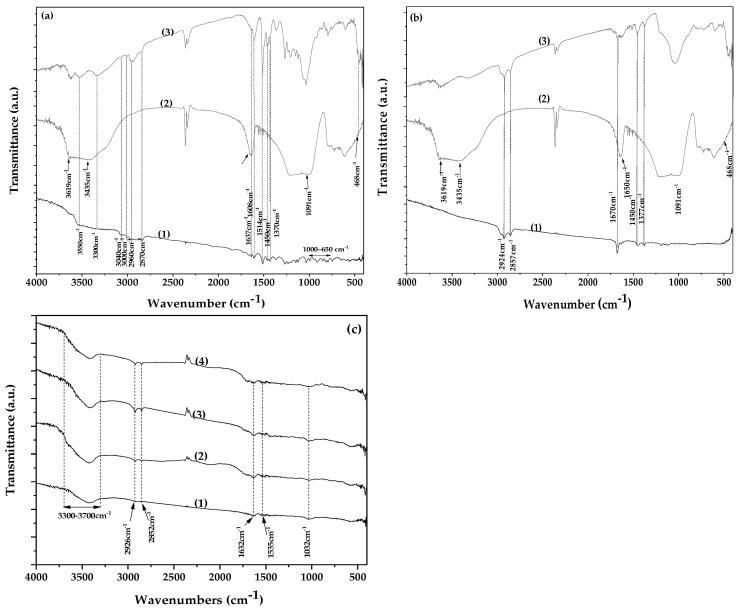
(**a**) FTIR spectra of (1) pure EG, (2) vacuum-dried NZ, and (3) EG@NZ nanohybrid, (**b**) FTIR spectra of (1) pure CT, (2) vacuum-dried NZ, and (3) CT@NZ nanohybrid, (**c**) FTIR spectra of (1) Gel/Gl25 matrix, (2) Gel/Gl25/NZ5, (3) Gel/Gl25/EG@NZ5, and (4) Gel/Gl25/CT@NZ5 composite films.

The FTIR spectrum of pure EG is presented as plot line (1) in Figure 4a. The characteristic absorption bands of EG are clearly observed in this spectrum. A broad band in the region of 3300–3550 cm^−1^ is attributed to O–H stretching vibrations [51]. The peaks at 3000 and 3040 cm^−1^ correspond to the stretching vibrations of the CH=CH–H groups, while the absorption bands in the 650–1000 cm^−1^ region are assigned to the bending vibrations of the same functional groups [51,52]. Additionally, peaks observed at 2870 and 2960 cm^−1^ are attributed to the symmetric and asymmetric stretching vibrations of methyl (CH_3_) groups, respectively. The corresponding symmetric and asymmetric bending vibrations of CH_3_ groups are observed at 1370 and 1450 cm^−1^ [51]. Finally, the peaks at 1514, 1608, and 1637 cm^−1^ are assigned to the aromatic C=C stretching vibrations of the EG molecule [51,53].

Plot line (2) in Figure 4a corresponds to the FTIR spectrum of pure NZ. The bands at 3619 and 3465 cm^−1^ are assigned to the O–H stretching vibrations, the band at 1650 cm^−1^ corresponds to the O–H bending vibration, the band at 1090 cm^−1^ is attributed to the Si–O stretching vibration, and the band at 468 cm^−1^ to the Si–O bending vibration [11].

Plot line (3) in Figure 4a shows the FTIR spectrum of the EG@NZ nanohybrid. This spectrum represents a combination of characteristic peaks from both pure NZ and EG, confirming the presence of both components and the successful adsorption of EG onto NZ. Furthermore, the simultaneous increase in the intensity of the O–H stretching vibrations of EG at 3300–3550 cm^−1^, coupled with a decrease in the NZ bending vibration at 1650 cm^−1^, suggests that EG adsorption occurs via interaction between the hydroxyl groups of both EG and NZ [54].

In Figure 4b, plot line (1) corresponds to the FTIR spectrum of pure CT. The peaks at 1377 cm^−1^ and 1450 cm^−1^ are assigned to the bending vibrations of methyl and methylene C–H bonds, respectively. The sharp peak at 1670 cm^−1^ corresponds to the stretching vibration of the aldehyde C=O bond in CT [55]. Peaks at 2857 cm^−1^ and 2924 cm^−1^ are attributed to the –CH_2_ and –CH_3_ stretching vibrations, respectively [56,57]. The FTIR spectrum of the CT@NZ nanohybrid (plot line (3)) exhibits a combination of peaks from both pure NZ and CT, confirming the presence of both materials and the adsorption of CT onto NZ. Notably, the hydroxyl group peaks of NZ at 3300–3550 cm^−1^ shift to lower wavenumbers, and the intensity of the aldehyde C=O stretching vibration of CT decreases, indicating an interaction between NZ’s hydroxyl groups and CT’s aldehyde group.

Overall, the FTIR results for both EG@NZ and CT@NZ nanohybrids confirm successful adsorption and interaction between the EG and CT molecules and NZ. This interaction appears stronger between the hydroxyl groups of NZ and the aldehyde carbonyl group of CT, compared to the interaction between NZ and the hydroxyl groups of EG.

Representative FTIR spectra of the films are presented in Figure 4c. The weak peak at 1032 cm^−1^ observed in all spectra corresponds to the O–H groups of glycerol. In gelatin, the peaks at 1535 cm^−1^ and 1632 cm^−1^ are attributed to the N–H bending vibrations of amide II and the C=O stretching vibrations of amide I, respectively, with the 1535 cm^−1^ peak also involving C–N stretching vibrations. The peaks at 2926 cm^−1^ and 2852 cm^−1^ are assigned to saturated C–H stretching vibrations, attributable to both gelatin and glycerol. The broad peak between 3300 and 3600 cm^−1^ corresponds to O–H stretching vibrations [58,59,60]. These characteristic peaks appear consistently in all four spectra shown in Figure 4c, indicating good blending of Gel with Gl. Notably, the characteristic peaks of the EOs and NZ are barely visible in the film spectra, which is expected due to their weak signals in the nanocomposite. Additionally, the absence of peak deconvolution or shift in the FTIR spectra shown in Figure 4c indicates successful physical dispersion of the nanohybrids within the polymer matrix and suggests no significant interactions between the nanohybrids and the Gel/Gl matrix, consistent with the XRD results.

### 2.4. SEM Morphology of Films

**Figure 5:**
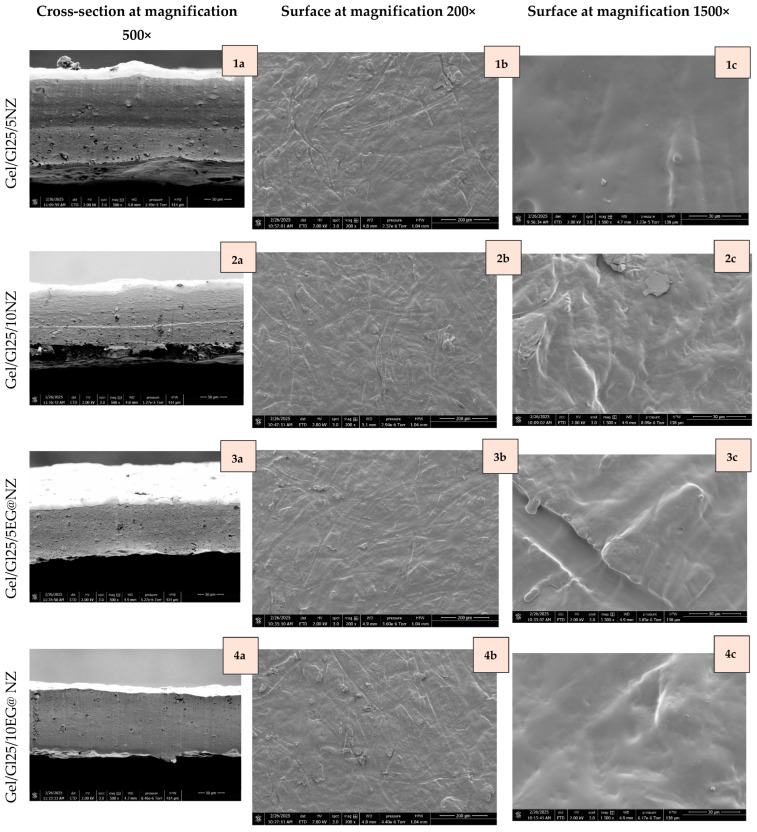

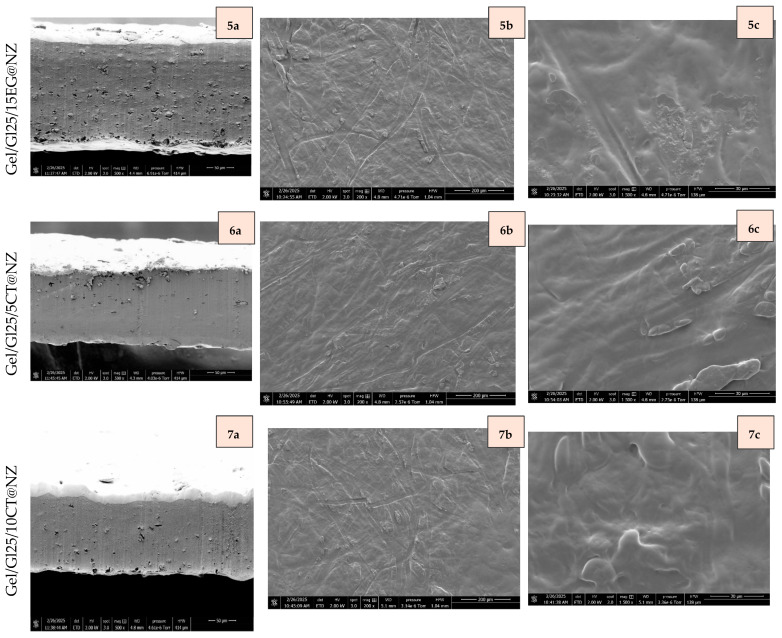
SEM images of film surfaces are presented for Gel/Gl25/5NZ (**1a**–**1c**), Gel/Gl25/10NZ (**2a**–**2c**), Gel/Gl25/5EG@NZ (**3a**–**3c**), Gel/Gl25/10EG@NZ (**4a**–**4c**), Gel/Gl25/15EG@NZ (**5a**–**5c**), Gel/Gl25/5CT@NZ (**6a**–**6c**), and Gel/Gl25/10CT@NZ (**7a**–**7c**). Cross-sectional images (**1a**–**7a**) are shown at 500× magnification, while surface morphology images (**1b**–**7b** and **1c**–**7c**) are presented at 200× and 1500× magnifications, respectively.

The morphological characteristics of all prepared Gel/Gl/xNZ, Gel/Gl/xEG@NZ, and Gel/Gl/xCT@NZ films were evaluated using scanning electron microscopy (SEM). Both the cross-sectional structures and surface morphologies were analyzed at magnifications of 500×, 200×, and 1500×, as presented in Figure 5.

SEM analysis of both cross-sectional and surface morphologies revealed that increasing the natural zeolite (NZ) content from 0.347 g (Gel/Gl/5NZ) to 0.733 g (Gel/Gl/10NZ) led to a less uniform film structure (Figure 5(1c) vs. Figure 5(2c)). Specifically, the surface of Gel/Gl/5NZ (Figure 5(1c)) appeared smoother at 1500× magnification, whereas Gel/Gl/10NZ (Figure 5(2c)) showed a noticeably rougher morphology, indicating that higher NZ loading reduces surface homogeneity. In contrast, differences between the two samples were less pronounced in the cross-sectional images (Figure 5(1a) vs. Figure 5(2a)) and at lower magnifications (Figure 5(1b) vs. Figure 5(2b)), suggesting that the structural impact of increased NZ content is more significant at the surface than within the internal matrix. These morphological changes likely stem from the high porosity and ion-exchange capacity of zeolites, which promote particle agglomeration and hinder uniform dispersion [61].

In contrast, incorporating EG and CT essential oils (EOs) into the NZ matrix significantly improved the dispersion of EG@NZ and CT@NZ particles, resulting in smoother and less rough film surfaces. This improvement was particularly evident in the cross-sectional images, where EO-loaded films displayed more homogeneous structures compared to films containing NZ alone (Figure 5(3a–3c)–(7a–7c) vs. Figure 5(1a) and Figure 5(2a)). A concentration-dependent enhancement in morphology was observed for both CT (Figure 5(3a) vs. Figure 5(4a)) and EG (Figure 5(5a) vs. Figure 5(6a) vs. Figure 5(7a)), indicating better nanohybrid integration at higher EO loadings. EOs are known to act as plasticizers in biopolymer films, enhancing flexibility and matrix homogeneity [62,63]. However, as Bonilla et al. reported, excessive oil content (e.g., 1% thyme nanoemulsion) can cause film heterogeneity due to emulsion instability during drying, underscoring the need to optimize EO concentration and incorporation methods [63].

Overall, these results suggest that higher EO concentrations promote more effective nanohybrid dispersion and integration within the film matrix, with no interfacial interactions, thereby improving not only antimicrobial activity but also the films’ structural integrity and surface texture.

### 2.5. Tensile Properties

**Figure 6:**
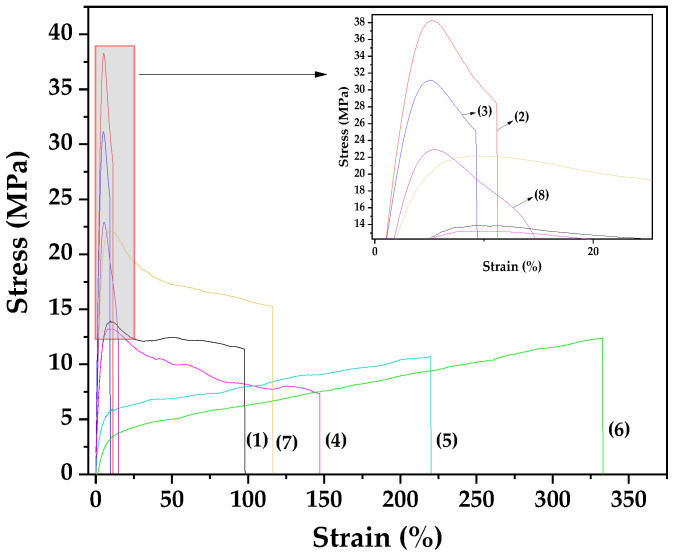
Stress–strain curves for the following films are presented: (1) Gel/Gl25, (2) Gel/Gl25/NZ5, (3) Gel/Gl25/NZ10, (4) Gel/Gl25/EG@NZ5, (5) Gel/Gl25/EG@NZ10, (6) Gel/Gl25/EG@NZ15, (7) Gel/Gl25/CT@NZ5, and (8) Gel/Gl25/CT@NZ10.

Figure 6 presents the stress–strain curves obtained from the tensile characterization of the prepared films. The derived values for elastic modulus (E), ultimate tensile strength (σ_uts_), and percentage elongation for all tested films are listed in Table 2 for comparison.

As shown in Table 2, the addition of 5 wt.% NZ to the Gel/Gl composite matrix significantly increases the elastic modulus and ultimate tensile strength (σ_uts_) by approximately 207% and 175%, respectively. At the same time, elongation at break drastically decreases from 97.73% to 11.16%. This indicates that the film becomes stronger (higher σ_uts_) and stiffer (higher elastic modulus), but also more brittle (lower elongation at break), confirming that NZ acts as a reinforcing agent in polymers and biopolymers, consistent with previous studies [15,64]. Increasing the NZ content to 10 wt.% results in a decline in all three tensile properties, suggesting that 5 wt.% NZ is the optimal loading for Gel/Gl/xNZ nanocomposite films.

In contrast, films containing EG@NZ and CT@NZ nanohybrids exhibit different behavior. At 5 wt.% EG@NZ loading, the elastic modulus and σ_uts_ remain nearly unchanged, while elongation increases by approximately 50%. Increasing the EG@NZ content to 10 and 15 wt.% progressively decreases the elastic modulus and σ_uts_, while elongation continues to rise. This indicates that EG@NZ results in softer (lower elastic modulus), weaker (lower σ_uts_), but tougher (higher elongation) films. In other words, the addition of EG@NZ imparts a plasticizing effect on the Gel/Gl matrix. Essential oils can exhibit such plasticizing behavior when incorporated into certain polymers or biopolymers, increasing flexibility while reducing material strength [65,66].

For films containing CT@NZ, the addition of 5 wt.% results in a significant increase in both elastic modulus and σ_uts_ without reducing elongation, producing a harder, stronger, and tougher film compared to the original Gel/Gl matrix. Increasing the CT@NZ content to 10 wt.% further enhances the elastic modulus and σ_uts_ but causes a notable decrease in elongation at break. This indicates that no plasticizing effect occurs with the CT@NZ nanohybrid; rather, its addition to the Gel/Gl matrix acts as reinforcement instead of a plasticizer.

Considering the release kinetics of EG and CT, along with the FTIR results of the EG@NZ and CT@NZ nanohybrids described above, it can be concluded that: (i) EG molecules are more weakly bonded to the NZ surface, reducing electrostatic interactions between Gel/Gl and NZ, thus promoting plasticization in Gel/Gl/xEG@NZ films [67], and (ii) CT molecules are more strongly bonded to NZ, with some fraction remaining unreleased, enhancing electrostatic interactions with Gel/Gl and improving tensile strength in Gel/Gl/xCT@NZ films.

### 2.6. Oxygen Barrier Properties of Gel/Gl/xNZ, Gel/Gl/xEG@NZ, and Gel/Gl/xCT@NZ Films

Table 3 lists the observed oxygen transmission rate (OTR) values and the calculated mean oxygen permeability (Pe_O2_) values for all tested Gel/Gl/xNZ, Gel/Gl/xEG@NZ, and Gel/Gl/xCT@NZ films, allowing for direct comparison. As shown in Table 3, all tested films exhibited zero oxygen transmission rate (OTR) values, indicating that the prepared films are completely impermeable to oxygen. It is well known that protein-based films, such as gelatin/sodium caseinate films, provide excellent oxygen barrier properties [68]. Also, it is well established that using low glycerol content (% wt.) and extrusion temperatures above 120 °C contribute to the high barrier properties of gelatin-based extruded films [20,69,70]. In this study, the subsequent compression molding process further enhanced the oxygen barrier performance of the gelatin films, achieving a complete oxygen barrier. Compression molding is widely recognized for improving the gas barrier properties of packaging films [71,72,73,74].

### 2.7. Antioxidant Activity of Gel/Gl/xNZ, Gel/Gl/xEG@NZ, and Gel/Gl/xCT@NZ Films

Table 3 presents the calculated mean EC_60_ values for all Gel/Gl/xEG@NZ and Gel/Gl/xCT@NZ films for comparison. EC_60_ values are preferred over EC_50_ when films exhibit high antioxidant capacity [75]. As observed, films based on EG@NZ demonstrated significantly higher antioxidant activity compared to those based on CT@NZ. This finding aligns with the release kinetics results discussed earlier, which showed that the EG@NZ nanohybrid releases a substantially greater amount of adsorbed EG than the CT@NZ nanohybrid due to weaker interactions between EG molecules and NZ compared to those between CT molecules and NZ.

### 2.8. Antibacterial Activity of Gel/Gl/xNZ, Gel/Gl/xEG@NZ, and Gel/Gl/xCT@NZ Films

**Figure 7:**
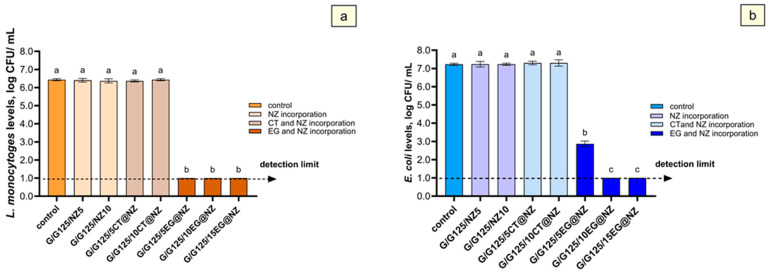
Mean populations of *Listeria monocytogenes* (**a**) and *Escherichia coli* (**b**) in the film samples are presented as log_10_-transformed values. Different letters (a, b, and c) indicate statistically significant differences between groups (*p* < 0.05). Error bars represent standard deviations. The detection limit for both bacterial populations was 1.0 log CFU/mL.

The antibacterial efficacy of the films was evaluated against *Listeria monocytogenes* (Figure 7a) and *Escherichia coli* (Figure 7b). The control group exhibited an average population of approximately 6.8 log CFU/mL for both pathogens. Similarly, films containing natural zeolite (NZ; Gel/Gl/NZ) at concentrations of 0.347 g (Gel/Gl/5NZ) and 0.733 g (Gel/Gl/10NZ) showed no effect on pathogen viability, confirming that natural zeolite acts primarily as a carrier for antimicrobial agents [76].

The antimicrobial activity of EG-loaded films against *Listeria monocytogenes* was significant (Figure 7), with all tested concentrations (0.347–1.160 g; Gel/Gl/5EG@NZ, Gel/Gl/10EG@NZ, and Gel/Gl/15EG@NZ) reducing bacterial counts below detection limits. This corresponds to a reduction of approximately 5.4 log CFU/mL compared to the control and natural zeolite groups. However, no clear dose-dependent effect was observed, as all concentrations reduced bacterial counts below detection limits (Figure 7).

In contrast, *Escherichia coli* showed a concentration-dependent response to EG-loaded films. The lowest concentration (0.347 g, Gel/Gl/5EG@NZ) caused a significant reduction of around 4.0 log CFU/mL compared to control and NZ-only groups (Gel/Gl/5NZ and Gel/Gl/10NZ). Higher concentrations (0.733 g, Gel/Gl/10EG@NZ and 1.160 g, Gel/Gl/15EG@NZ) further reduced *E. coli* counts below detection limits, with reductions up to approximately 6.2 log CFU/mL.

Films containing CT (0.347 g, Gel/Gl/5CT@NZ and 0.733 g, Gel/Gl/10CT@NZ) exhibited no antimicrobial effect against either *L. monocytogenes* or *E. coli*, indicating that these CT concentrations were not sufficient to inhibit bacterial growth. The lower antimicrobial efficacy of CT can be attributed to its comparatively weaker activity, as supported by previously reported higher minimal bactericidal concentration (MBC) and minimum inhibitory concentration (MIC) values for CT compared to EG. For example, MIC and MBC values against *E. coli* STEC O26 are 0.71 and 1.26 mg/mL for CT, respectively [77,78] while against *Aspergillus niger*, MIC values are 0.17 mg/mL for CT compared to 0.06 mg/mL for EG [79].

Overall, *L. monocytogenes* was more susceptible to EG-functionalized films (Figure 7a) than *E. coli* (Figure 7b). The most effective formulation, Gel/Gl/10EG@NZ, completely inhibited growth of both pathogens. At lower EG concentrations (Gel/Gl/5EG@NZ), *L. monocytogenes* exhibited a 3.5 log CFU/mL reduction, while *E. coli* showed only a 0.8 log CFU/mL reduction, indicating greater resistance of *E. coli* to EG.

Finally, the generally higher antibacterial activity observed in EG-based films aligns with the release kinetics results, which show higher EG release rates due to its weaker interaction with the NZ matrix compared to CT.

### 2.9. Packaging Preservation Test

#### 2.9.1. Total Viable Count (TVC)

Total viable count (TVC) is a standard established quality parameter for the evaluation of the safety of meat [80,81]. Table 4 presents the mean total viable counts (TVC) of fresh pork ham slices stored at 4 ± 1 °C for twenty-six days. The tested samples include those wrapped with Gel/Gl/15EG@NZ and Gel/Gl/10CT@NZ active films used as extra active pads, alongside control samples without any additional active pads.

As shown by the TVC values listed in Table 4, the following observations can be made: (i) TVC values of fresh pork ham slices wrapped with commercial film increased steadily over the 26-day storage period and exceeded the acceptance limit of 7 log CFU/g after the 24th day of preservation [82], (ii) TVC values of fresh pork ham slices with Gel/Gl/15EG@NZ and Gel/Gl/10CT@NZ active pads increased at a slower rate compared to those wrapped with commercial film, (iii) in both cases, TVC values remained below the acceptance limit of (7 log CFU/g) throughout the 26-day storage period, (iv) the Gel/Gl/15EG@NZ active pad showed the most effective inhibition of microbial growth, with the lowest TVC increase rate, and (v) notably the Gel/Gl/15EG@NZ film maintained stable TVC levels from the 10th day of storage onward.

Gel-based films are well known for their barrier, antimicrobial, and antioxidant properties, offering a promising solution for meat preservation by extending shelf life and maintaining product quality [83,84]. When combined with other biopolymers such as chitosan or pullulan or bioactive agents like nisin, cathegin, or EG, Gel-based films have been reported to further enhance preservation of pork and beef meat products [83,84,85,86]. The current findings are consistent with previous studies on Gel-based active films, but to our knowledge, this is the first report demonstrating the application of such films as active pads specifically for the preservation of pork ham slices.

#### 2.9.2. TBA

The obtained TBA values for fresh pork ham slices stored with Gel/Gl/15EG@NZ and Gel/Gl/10CT@NZ active films, as well as for control samples (without active pads), during storage at 4 ± 1 °C for twenty-six days are listed in Table S5 in the Supplementary Materials.

According to Table S5, all pork ham slices maintained low TBA values throughout the storage period, with no significant differences observed among the different groups. This suggests that the nitrate/nitrite salts added during industrial pork processing effectively protected lipids from oxidative degradation, and no additional antioxidant effect from either EG@NZ or CT@NZ active pads was observed. These TBA results appear to contrast with the TVC findings presented earlier. This disrepancy likely arises from the fundamentally different mechanisms of antibacterial and antioxidant action [87,88,89]. Antibacterial agents disrupt bacterial cell membranes, primarily through hydrophobic interactions with membrane lipids, leading to metabolic damage and cell death [90]. In contrast, antioxidants function by neutralizing free radicals, particularly reactive oxygen species, which cause oxidative damage to lipids and other biomolecules [87]. Nitrate/nitrite salts are known to exhibit antioxidant properties, likely due to their conversion into nitric oxide (NO), which scavenges reactive oxygen and nitrogen species [91,92]. It is therefore plausible that nitrate/nitrite salts more effectively neutralize oxygen radicals than EG or CT molecules, making their contribution dominant in antioxidant protection. This interpretation aligns with the findings of Oliveira et al. who reported that EO addition, in combination with 200 mg/kg sodium nitrite, did not improve lipid oxidation stability [93]. In fact, they observed a potential antagonistic interaction, suggesting that nitrite may react with phenolic compounds, thereby inhibiting their antioxidant effect [93]. Furthermore, Candido Júnior et al. proposed that nitration of EG could lead to the formation of strong internal bonds, diminishing its hydrogen-donating ability and thus impairing its antioxidant effectiveness [94]. Moreover, a notable discrepancy was observed between the high antioxidant activity measured by DPPH-radical-scavenging assays and the comparatively moderate inhibition of lipid oxidation reported in the TBA assay. This discrepancy may be related to the release kinetics of the encapsulated EG and CT molecules, their diffusion limitations within the gelatin matrix, or the differing sensitivities of these assays to free radicals versus lipid peroxidation pathways [11,95,96].

## 3. Conclusions

In this study, the successful synthesis and characterization of novel EG@NZ and CT@NZ nanohybrids were demonstrated, along with their effective incorporation into a gelatin/glycerol (Gel/Gl) matrix via an extrusion–compression molding process to fabricate high-barrier active films (Gel/Gl/xEG@NZ and Gel/Gl/xCT@NZ). Release kinetics revealed that both nanohybrids achieved an essential oil loading of 58 wt%. EG@NZ released up to 95% of the adsorbed EG, while CT@NZ released up to 44%, suggesting a fraction of CT molecules are more strongly bound to the NZ surface, likely through hydrogen bonding involving CT’s aldehyde group and NZ’s hydroxyl groups. This was supported by desorption energy (E_0,des_) calculations, which indicated stronger intermolecular interactions in the EG@NZ system. As a result, lower desorption rate constants (k_2_) were observed for EG@NZ compared to CT@NZ.

XRD and SEM analyses confirmed successful EO adsorption onto NZ and evidenced structural changes upon hybrid formation. FTIR spectra suggested interaction of EG with NZ through OH group hydrogen bonding, while CT showed interactions involving its aldehyde group. EG@NZ nanohybrids were successfully dispersed into the Gel/Gl matrix at loadings of 5, 10, and 15 wt%., while CT@NZ nanohybrids were effectively incorporated up to 10 wt%. XRD and FTIR analyses further confirmed the uniform dispersion of nanohybrids within the films. SEM imaging revealed that increasing NZ content reduced film uniformity, whereas EO incorporation improved dispersion and reduced surface roughness.

Tensile property analysis indicated that EG, due to weaker bonding with NZ, reduced electrostatic interactions with the matrix and promoted plasticization in Gel/Gl/xEG@NZ films. In contrast, CT’s stronger bonding enhanced matrix–filler interactions, improving tensile strength in Gel/Gl/xCT@NZ films. Both active films exhibited zero oxygen permeability. Antioxidant and antibacterial assays showed that Gel/Gl/xEG@NZ films demonstrated significantly higher activity—measured by EC_60_ values and effectiveness against *Listeria monocytogenes* and *Escherichia coli*—than Gel/Gl/xCT@NZ films. These findings are consistent with the greater release capacity of EG compared to CT from the NZ matrix due to the weaker molecular interactions between EG and NZ.

Application trials using fresh pork ham slices demonstrated the potential of these films as active packaging pads. Both Gel/Gl/15EG@NZ and Gel/Gl/10CT@NZ films maintained lower total viable count (TVC) values over 26 days of storage at 4 ± 1 °C compared to samples wrapped in commercial film without active pads. Notably, the Gel/Gl/15EG@NZ film showed the most promising performance, maintaining a consistently low TVC growth rate after the 10th day of storage. A future direction for this study could be the evaluation of these films’ performance in preserving other perishable items, such as fruits, meats, or bakery products, under various storage conditions. Such investigations would provide practical validation and enhance their relevance for industrial adoption.

Overall, this study links the molecular interactions of EG and CT molecules and their release kinetics with the observed tensile, antioxidant, and antibacterial properties of the films. Consequently, it offers valuable guidance for the development and material design of essential-oil-based active packaging films.

## 4. Materials and Methods

### 4.1. Materials

The materials used for the preparation and characterization of the films were gelatin, glycerol, eugenol, citral, zeolite powder, 2,2-diphenyl-1-picrylhydrazyl (DPPH), and ethanol. Gelatin type A with a catalog number AC611995000 and CAS 9000-70-8 was purchased from Thermos Scientific Chemicals (Thermo Fisher Scientific, 168 Third Avenue, Waltham, MA USA 02451). Glycerol 99% with CAS number 56-81-5, was purchased from Labchem (Zelienople, PA, USA). Eugenol (2-methoxy-4-(2-propenyl) phenol, 4-allyl-2-methoxyphenol, 4-allylguaiacol) with CAS number 97-53-0 and citral, lemonal (3,7-dimethyl-2,6-octadienal) with CAS number 5392-40-5 and 2,2-diphenyl-1-picrylhydrazyl (DPPH) with CAS number 1898-66-4 were purchased from Sigma-Aldrich (Darmstadt, Germany). Zeolite powder 100 gr with product code 102.057.004 was purchased from Health Trade (Patras, Greece). Ethanol ROTIPURAN^®^ ≥99.8%, p.a. with CAS number 64-17-5 was purchased from Carl Roth (Karlsruhe, Germany).

### 4.2. Preparation of EG@NZ and CT@NZ

The preparation of EG@NZ and CT@NZ nanohybrids was conducted using a recently reported vacuum-assisted adsorption process [95]. Briefly, 2 g of as-received NZ was placed in a round-bottom glass flask and subjected to heating at 100 °C under vacuum for 15 min to eliminate any adsorbed moisture. Following the drying process, EG and CT were gradually added dropwise to the dried NZ under continuous stirring to facilitate uniform adsorption. The resulting EG@NZ and CT@NZ nanohybrids were then collected and weighed. The calculated loading content of EG and CT on their respective nanohybrids was approximately 58 wt%. each.

### 4.3. Preparation of Gel/Gl, Gel/Gl/xNZ, Gel/Gl/xEG@NZ, and Gel/Gl/xCT@NZ Membranes

The Gel/Gl/xNZ, Gel/Gl/xEG@NZ, and Gel/Gl/xCT@NZ films were fabricated using the extrusion method, utilizing a twin-screw mini lab extruder (Haake Mini Lab II, Thermo Scientific, ANTISEL, S.A., Athens, Greece). The “x” factor in the code names of the membranes signifies the percentage of the added nanostructure or nanocomposite in the mix. To develop the “blank” Gel/Gl membrane, a mixture of 4 g gelatin, 1 g glycerol, and 1.6 g of water was extruded at 110 °C and 250 rounds per minute (rpm) for 3 min (min). These extrusion conditions were consistently applied to all film formulations discussed in this study. The extrudate threads were then molded into films using heating platens (Specac Atlas™ Series Heated Platens, Specac, Orpinghton, UK) at 110 °C under 1tn pressure for 2 min. The obtained films had an average diameter of 10 cm and a thickness ranging from 0.10–0.15 mm. For the modified films, the same processing steps were followed, with compositional differences introduced in the initial blend. Specifically, the Gel/Gl/5NZ and Gel/Gl/10NZ films were prepared by adding to the “blank” composition 0.347 g and 0.733 g of NZ, respectively. The Gel/Gl/5EG@NZ, Gel/Gl/10EG@NZ, and Gel/Gl/15EG@NZ were prepared by adding to the “blank” composition 0.347 g, 0.733 g, and 1.160 g of EG@NZ, respectively. Likewise, the Gel/Gl/5CT@NZ and Gel/Gl/10CT@NZ were prepared by adding 0.347 g and 0.733 g of CT@NZ, respectively. The detailed masses of the components and the extruding conditions are shown in Table 5.

### 4.4. Physicochemical Characterization of EG@NZ and EG@NZ Nanohybrids

The obtained EG@NZ and EG@NZ nanohybrids as well as pure NZ were characterized using X-ray diffraction (XRD), Fourier-transform infrared spectroscopy (FTIR), and scanning electron microscopy (SEM) following the procedures outlined in the Supplementary Materials. Furthermore, the desorbed amounts of eugenol (EG) and citral (CT) from the NZ surface, along with their release rates, were determined through desorption kinetic experiments conducted on both EG@NZ and CT@NZ nanohybrids. These experiments were performed using a moisture analyzer (AXIS AS-60 AXIS Sp. z o.o. ul. Kartuska 375b, 80–125 Gdańsk, Poland) in accordance with the methodology described in the Supplementary Materials.

### 4.5. Physicochemical Characterization of Gel/Gl/xNZ, Gel/Gl/xEG@NZ, and Gel/Gl/xCT@NZ Films

The obtained Gel/Gl/xNZ (x = 5, 10), Gel/Gl/xEG@NZ (x = 5, 10, 15), and Gel/Gl/xCT@NZ (x = 5, 10) films were physicochemically characterized with XRD analysis, FTIR spectroscopy, and SEM analysis by following the methodology and instrumentation given in the Supplementary Materials file.

### 4.6. Packaging Properties of Gel/Gl/xNZ, Gel/Gl/xEG@NZ, and Gel/Gl/xCT@NZ Films

Tensile and oxygen barrier properties of all obtained films were determined according to the ASTM D638 [97] and ASTM D3985 [98] methods, respectively, by following the instrumentation and methodology described in the Supplementary Materials. The in vitro antioxidant and antibacterial activities of the films were also assessed following the experimental protocols described comprehensively in the Supplementary Materials file.

### 4.7. Packaging Preservation Test of Fresh Pork Ham Slices with Gel/Gl/15EG@NZ, Gel/Gl/10CT@NZ Films Applied as Extra Active Pads

For the packaging preservation test of fresh pork ham, the Gel/Gl/15EG@NZ and Gel/Gl/10CT@NZ active films were selected as the optimal formulations. Fresh pork ham was kindly provided by the meat processing company Ayfantis (Agrinio, Greece). The meat was aseptically sliced and initially wrapped in the commercial Ayfantis packaging paper. The “ready-to-eat” pork ham had undergone pasteurization for 10 min at 72 °C, with nitrate and nitrite salts added in accordance with the European Food Safety Authority (EFSA) Directive 95/2/EC, at concentrations of 4.5 mg/kg for nitrite ions and 9.6 mg/kg for nitrate ions [99,100,101].

**Figure 8:**
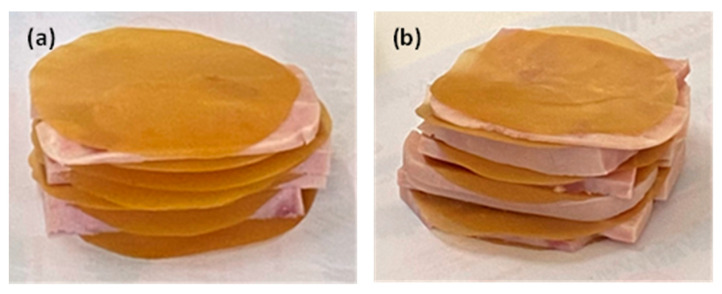
Fresh pork ham slices with (**a**) Gel/Gl/15EG@NZ and (**b**) Gel/Gl/10CT@NZ films inserted as active pads between the slices.

Three sample groups were prepared: (i) control samples, consisting of sliced pork ham wrapped only in commercial Ayfantis paper, (ii) sliced pork ham with a Gel/Gl/15EG@NZ active film inserted as an additional active pad and then wrapped in commercial paper, (iii) sliced pork ham with a Gel/Gl/10CT@NZ active pad similarly applied and wrapped (see Figure 8).

All samples were stored under refrigerated conditions (4 ± 1 °C, LG GC-151SA, Weybridge, UK) in the dark. Sampling was carried out on the 2nd, 4th, 6th, 10th, 14th, 18th, 22nd, and 26th day of storage. Total viable count (TVC) and thiobarbituric acid reactive substances (TBARS) values were measured at each time point following the analytical protocols detailed in the Supplementary Materials.

### 4.8. Statistical Analysis

k_2_, q_e_, E^0^_,des_, EC_60_, elastic modulus (E), ultimate strength (σ_uts_), % elongation at break (%ε), biocompatibility, antibacterial, total variable count (TVC), and TBA mean values as well as their stadard deviation were calculated. These properties were further subjected to statistical analysis using one-way analysis of variance (ANOVA) followed by Tukey’s honestly significant difference (HSD) post hoc test to identify statistically significant differences between the mean values. A significance threshold of *p* < 0.05 was adopted for all comparisons. Each measurement was based on five to seven independent replicates per film type (Gel/Gl/xNZ, Gel/Gl/xEG@NZ, and Gel/Gl/xCT@NZ). Statistical analyses were performed using SPSS software (version 28.0; IBM Corp., Armonk, NY, USA).

## Figures and Tables

**Table 1:** Calculated k_2_, q_e_, mean values from EG, CT desorption kinetic plots for both EG@NZ and CT@NZ nanohybrids.

Sample Code	Temp. (°C)	k_2_ × 10^−4^ (s^−1^)	% q_e_	R^2^
EG@NZ	70	0.217 ± 0.0358	90 ± 1	0.964 ± (2.8 × 10^−4^)
	90	1.470 ± 0.2400	93 ± 1	0.934 ± (4.0 × 10^−4^)
	110	10.900 ± 0.5000	95 ± 1	0.931 ± (2.4 × 10^−4^)
CT@NZ	70	12.700 ± 0.5000	33 ± 2	0.993 ± (1.2 × 10^−5^)
	90	25.000 ± 0.8000	36 ± 3	0.994 ± (3.3 × 10^−7^)
	110	30.300 ± 0.1000	44 ± 7	0.990 ± (1.3 × 10^−6^)

where k_2_ (s^−1^) is the desorption rate constant used in the normalized expression for estimating qₜ (Equation (S2), Supplementary Materials), qₜ is the normalized fraction of EOs desorbed at time t, and qₑ is the normalized fraction of the total desorbed EO amount, with values ranging between 0 and 1 (0 < qₜ, qₑ < 1).

**Table 2:** Calculated mean values of Elastic Modulus, ultimate strength (σ_uts_), and elongation at break for all tested films.

Specimen	Elastic Modulus (Ε) (MPa)	Ultimate Strength (σ_uts_) (MPa)	%Elongation (%ε)
Gel/Gl25	393.94 ± 56.01 ^C^	13.90 ± 2.11 ^A^	97.73 ± 54.64 ^D^
Gel/Gl25/NZ5	1209.62 ± 109.94 ^A^	38.25 ± 2.98 ^A^	11.16 ± 6.68 ^G^
Gel/Gl25/NZ10	1084.73 ± 193.81 ^A^	31.17 ± 2.86 ^B^	9.33 ± 4.31 ^G^
Gel/Gl25/EG@NZ5	384.40 ± 104.99 ^C^	13.21 ± 3.50 ^A^	147.05 ± 84.19 ^B^
Gel/Gl25/EG@NZ10	112.63 ± 5.07 ^D^	5.97 ± 0.61 ^C^	219.90 ± 41.06 ^A^
Gel/Gl25/EG@NZ15	71.15 ± 17.96 ^D^	4.76 ± 1.11 ^C^	332.93 ± 39.94 ^A^
Gel/Gl25/CT@NZ5	610.38 ± 101.72 ^B^	22.10 ± 6.01 ^B^	116.18 ± 19.15 ^C^
Gel/Gl25/CT@NZ10	741.94 ± 62.61 ^B^	22.92 ± 1.51 ^B^	14.84 ± 2.84 ^E^

Different capital letters in the same column indicate significant differences between treatments (Tukey HSD, *p <* 0.05). See also Table S1 in the Supplementary Materials file.

**Table 3:** Oxygen transmission rate (OTR) mean values, as well as the calculated oxygen permeability Pe_O2_ mean values of all tested films.

	Thickness (mm)	OTR (mL·m^−2^·day^−1^)	P_eO2_ (cm^2^·s^−1^) × 10^−9^	EC_60_ (mg/L)
Gel/Gl	0.08 ± 0.01	0	0	-
Gel/Gl/5NZ	0.12 ± 0.04	0	0	-
Gel/Gl/10NZ	0.15 ± 0.01	0	0	-
Gel/Gl/5EG@NZ	0.13 ± 0.01	0	0	7.4 ± 0.2 ^B^
Gel/Gl/10EG@NZ	0.14 ± 0.02	0	0	8.9 ± 0.3 ^A^
Gel/Gl/15EG@NZ	0.09 ± 0.01	0	0	8.2 ± 0.1 ^A^
Gel/Gl/5CT@NZ	0.09 ± 0.01	0	0	205 ± 0.3 ^D^
Gel/Gl/10CT@NZ	0.08 ± 0.01	0	0	7.4 ± 0.2 ^B^

Different capital letters in the same column indicate significant differences between treatments (Tukey HSD, *p* < 0.05).

**Table 4:** TVC mean values of fresh pork ham slices packaged with Gel/Gl/15EG@NZ, Gel/Gl/10CT@NZ active films used as extra active pads as well as control samples without active pads, during 26 days of storage at 4 ± 1 °C.

**Sample Code**	**logCFU/g**			
	**Day** **0**	**Day 2**	**Day 4**	**Day 6**
Control	0.47 ± 0.06 ^aA^	1.53 ± 0.75 ^aA^	1.98 ± 0.18 ^aA^	2.19 ± 0.16 ^aA^
Gel/Gl/10CT@NZ	0.47 ± 0.06 ^aA^	2.26 ± 0.20 ^abB^	1.37 ± 0.35 ^cA^	1.94 ± 0.52 ^bB^
Gel/Gl/15EG@NZ	0.47 ± 0.06 ^aA^	0.56 ± 0.15 ^bA^	0.97 ± 0.15 ^cB^	2.59 ± 0.17 ^cc^
**Sample Code**	**logCFU/g**			
	**Day 10**	**Day 14**	**Day 18**	**Day 22**
Control	1.03 ± 0.16 ^aA^	3.02 ± 0.43 ^aA^	4.85 ± 0.04 ^aA^	5.72 ± 0.17 ^aA^
Gel/Gl/10CT@NZ	2.85 ± 0.20 ^aB^	2.94 ± 0.52 ^abB^	3.24 ± 0.27 ^cB^	4.50 ± 0.32 ^bB^
Gel/Gl/15EG@NZ	1.13 ± 0.15 ^aB^	1.13 ± 0.15 ^aB^	1.33 ± 0.14 ^bB^	1.06 ± 0.12 ^cc^
**Sample Code**	**logCFU/g**			
	**Day 26**			
Control	7.69 ± 0.09 ^aA^			
Gel/Gl/10CT@NZ	5.43 ± 0.12 ^abB^			
Gel/Gl/15EG@NZ	1.13 ± 0.15 ^bc^			

Different capital letters in the same column indicate significant differences between treatments on the same day (Tukey HSD, *p* < 0.05). Different lowercase letters in the same row indicate significant differences between storage days for the same treatment. See also Table S5 in the Supplementary Materials file.

**Table 5:** Sample names of the films, weighed masses of their composites (gelatin, glycerol, H_2_O, NZ, EG@NZ and CT@NZ), and twin extruder operating conditions (temperature, rotating speed, and operating time).

Sample Name	Gelatin (g)	Glycerol (g)	H_2_O (g)	NZ (g)	EG@NZ (g)	CT@NZ (g)
Gel/Gl	4	1	1.6	-	-	-
Gel/Gl/5NZ	4	1	1.6	0.347	-	-
Gel/Gl/10NZ	4	1	1.6	0.733	-	-
Gel/Gl/5EG@NZ	4	1	1.6	-	0.347	-
Gel/Gl/10EG@NZ	4	1	1.6	-	0.733	-
Gel/Gl/15EG@NZ	4	1	1.6	-	1.160	-
Gel/Gl/5CT@NZ	4	1	1.6	-	-	0.347
Gel/Gl/10CT@NZ	4	1	1.6	-	-	0.733

## Data Availability

The datasets generated for this study are available on request to the corresponding author.

## Supplementary Materials

The following supporting information can be downloaded at: https://www.mdpi.com/article/10.3390/gels11070518/s1, Figure S1: Linear plots used for the calculation of average values of EC_50_ and EC_60_; Table S1: Tensile properties statistical analysis results; Table S2: Experimental data used for the calculation of obtained average EC_60_ values; Table S3: Statistical analysis results of EC_60_ values; Table S4: Statistical analysis results of antibacterial activity results; Table S5: Calculated TBA mean values of fresh pork ham slices with Gel/Gl/15EG@NZ, Gel/Gl/10CT@NZ active films applied as extra active pads as well as fresh pork ham slices without extra active pads (control sample) during the twenty-six days of storage at 4 ± 1 °C. Table S6: Statistical analysis results of TBA results; Table S7: Statistical analysis results of TVC [102,103,104,105,106,107,108,109,110,111,112,113,114].
