# Supplementary material for: Eugenol@natural Zeolite vs. Citral@natural Zeolite Nanohybrids for Gelatin-Based Edible-Active Packaging Films
Source: Gels. 2025 Jul 3;11(7):518. doi: 10.3390/gels11070518 (PMC12294803; doi:10.3390/gels11070518)
Supplement: Supplementary file 1 [file gels-11-00518-s001.zip › gels-3701215-supplementary.pdf]

Supplementary material for:

Eugenol@natural zeolite vs citral@natural zeolite nanohybrids for gelatine based edible-active packaging films

Achilleas Kehagias [1], Areti A. Leontiou[1], Yelyzaveta K. Oliinychenko[2], Alexandros Ch. Stratakos[2], Nikolaos Chalmpes[3*], Konstantinos Zaharioudakis[1], Katerina Katerinopoulou[1], Nikolaos D. Andritsos[1], Charalampos Proestos[4], Constantinos E. Salmas[5,*], and Aris E. Giannakas [1,*]

1 Department of Food Science and Technology, University of Patras, 30100 Agrinio, Greece; up1110842@upatras.gr (A.K.), aleontiu@upatras.gr (A.L.); zacharioudakis.k@upatras.gr (K.Z.); nandritsos@upatras.gr (N.Δ.A.) akaterin@upatras.gr (K.K.)

2 School of Applied Sciences, College for Health, Science and Society, University of the West of England, Coldharbour Ln, Bristol, BS16 1QY, United Kingdom. Yelyzaveta2.Oliinychenko@live.uwe.ac.uk(Y.O.) Alexandros.stratakos@uwe.ac.uk (A.C.S)

3 Department of Materials Science and Engineering, Cornell University, Ithaca, New York 14850, United States; nc427@cornell.edu (N.C.)

4 Laboratory of Food Chemistry, Department of Chemistry, National and Kapodistrian University of Athens Zografou, 15771 Athens, Greece; harpro@chem.uoa.gr (C.P.)

5 Department of Material Science and Engineering, University of Ioannina, 45110 Ioannina, Greece;

* Correspondence: ksalmas@uoi.gr (C.E.S.), agiannakas@upatras.gr (A.E.G.);

X-Ray Diffraction (XRD) studies

Obtained EG@NZ and CT@NZ nanohybrids pure NZ as well as all obtained Gel/Gl/xNZ, Gel/Gl/xEG@NZ and Gel/Gl/xCT@NZ films and pure Gel/Gl film were characterized with X-Ray Diffraction (XRD) analysis using a Brüker XRD D8 Advance diffractometer (Brüker, Analytical Instruments, S.A., Athens, Greece).

Fourier Transform Infrared Spectroscopy (FTIR) studies

FTIR spectra of pure EG, CT oils, EG@NZ and CT@NZ nanohybrids pure NZ as well as all obtained Gel/Gl/xNZ, Gel/Gl/xEG@NZ and Gel/Gl/xCT@NZ films and pure Gel/Gl film were recorded using an FT/IR-6000 JASCO Fourier-transform spectrometer (JASCO, Interlab, S.A., Athens, Greece). Measurements were carried out using the KBr (0.5 %wt. to 1 %wt.) tablet technique. The spectra recorded over the wavenumber range from 4000 to 400 cm^{-1} at a resolution of 4 cm^{-1} and 64 scans were averaged to reduce noise.

Scanning Electron Microscopy (SEM) studies

The surface morphology of the film samples was examined using a FEI Quanta 650 Field Emission Scanning Electron Microscope (FE-SEM, Thermo Fisher Scientific, Hillsboro, OR, USA). For analysis, the films were sectioned into longitudinal and cross-sectional fragments and mounted onto aluminium stubs using double-sided carbon conductive tape to ensure stability during imaging.

To enhance conductivity and mitigate charging effects, all samples were sputter-coated with a thin (~10 nm) layer of gold using a Quorum Q150R ES sputter coater (UK) in an argon atmosphere. The coating was applied for 10 minutes at a current of 20 mA, ensuring a uniform conductive layer across the sample surfaces. SEM images were analysed at magnifications of 200×, 500×, and 1,500×, providing both a broad overview of surface morphology and a detailed examination of microstructural features. A secondary electron (SE) detector was utilized to enhance topographical contrast, enabling detailed visualisation of surface texture, microstructural composition, and morphological characteristics of the film.

EG release kinetic studies of EG@Lap and EG@NZ nanohybrids

Approximately 100 mg of each nanohybrid was placed in the moisture analyzer and its weight was recorded (in triplicates) as a function of time (NZ) at 70, 90, and 110 °C. From the obtained NZ vs t measurements, the normalized values of the fraction q_t=(1−NZ/m_0) were calculated and plotted as a function of time. The plots were fitted using the well-known pseudo-second-order adsorption-desorption equation [1,2]. For process order, n=2 the overall normalized mass balance is given by:

$$\frac{dq_t}{dt} = k_2 * (q_e - q_t)^2 \qquad \text{(S1)}$$

where k_2 is the rate constant of the pseudo-second-order kinetic model (s^{-1}), qt is the desorbed fraction capacity at time t, q_e=(1-m_e/m_0) is the maximum desorbed fraction capacity at equilibrium, m_0 is the initial EOs loading into the nanohybrid, and NZ is the EOs amount remaining in the nanohybrid at time t. By integrating equation (S1) we achieve the pseudo-second-order kinetic model:

$$q_t = (1 - \frac{m_t}{m_0}) = \frac{q_e^2 * k_2 * t}{q_e * k_2 * t + 1} \qquad \text{(S2)}$$

The initial release rate can be computed via the equation (S1) and for t=0 (i.e., q_t=0). Thus:

$$r_i = \left.\frac{dq_t}{dt}\right|_{t=0} = k_2 * q_e^2 \qquad \text{(S3)}$$

From the best-fitted plots, the k_2 and q_e values were calculated. Using the estimated k_2 parameter the ln(k_2) term was calculated and plotted as a function of (1/T) to determine the desorption energy (E^0_{des}) according to the Arrhenius equation and the theory presented in detail in [3–5]:

$$k_2 = k_0 \cdot e^{-\frac{E^0_{des}}{R \cdot T}} \qquad \text{(S4)}$$

and its linear transformed type:

$$ln(k_2) = ln(k_0) - \frac{E^0_{des}}{R \cdot T} \qquad \text{(S5)}$$

where k_2 is the rate constant of the pseudo-second order kinetic model (s^{-1}), E^0_{des} is the desorption activation energy, and A is the Arrhenius constant.

Tensile properties of Gel/Gl/xNZ, Gel/Gl/xEG@NZ and Gel/Gl/xCT@NZ films

Tensile properties of Gel/Gl/xNZ, Gel/Gl/xEG@NZ and Gel/Gl/xCT@NZ films as well as pure Gel/Gl film were determined according to the American Society for Testing and Materials (ASTM) D638 method, by employing a Shimadzu AG-Xplus (5 kN), instrument (Shimadzü, Kyoto, Japan) and the methodology described in detail recently [6].

Oxygen barrier properties of Gel/Gl/xNZ, Gel/Gl/xEG@NZ and Gel/Gl/xCT@NZ films

Oxygen barrier properties of Gel/Gl/xNZ, Gel/Gl/xEG@NZ and Gel/Gl/xCT@NZ films as well as pure Gel/Gl film were determined according to ASTM D 3985 at 23 °C and 0 %RH using an oxygen permeation analyzer (O.P.A., 8001, Systech Illinois Instruments Co., Johnsburg, IL, USA)

by following the methodology described in detail recently [6]. The obtained oxygen transmission rate (OTR cc/m^2/day) values were transformed to oxygen diffusion coefficient values (P_{eO2}) using the methodology described in detail elsewhere [7].

In Vitro Antioxidant Activity Determination of Gel/Gl/xNZ, Gel/Gl/xEG@NZ and Gel/Gl/xCT@NZ films

For the CT based active films the concentration required to obtain a 50% antioxidant effect (EC_{50}) was calculated according to the methodology described recently [6] while for the EG based active films the concentration required to obtain a 60% antioxidant effect (EC_{60}) was calculated according to the methodology described recently by Karabagias et al. [8].

In detail for the preparation of [DPPH$^\bullet$] free radical standard solutions, 0.0212 g of [DPPH$^\bullet$] free radical was dissolved in 250 mL of methanol to obtain a 2.16 mM (mmol/L) methanolic solution. Next, the flask was vortexed under dark conditions, and its pH (Milwaukee MW102-FOOD PRO+ 2-in-1 pH and Temperature Meter) was measured to ensure its neutrality (7.02 ± 0.01). Finally, the solution was placed in a refrigerator at 4 ± 1 °C under dark conditions for stabilization.

For the preparation of a [DPPH$^\bullet$] free radical calibration curve, 2.16 mM (mmol/L) methanolic solution of [DPPH$^\bullet$] free radical was diluted by adding appropriate volumes of methanol to obtain concentrations of 10, 20, 30, 40, and 50 mg/L, and their absorbance was measured with a SHIMADZU UV-1900 UV/VIS Spectrometer at 517 nm. The calibration curve of absorbance (y) versus the concentration (x) of [DPPH$^\bullet$] free radical was expressed by the following equation:

$$y = 0.0388x + 0.015;\ R^2 = 0.9994 \qquad \text{(S6)}$$

For the determination of the concentration required to obtain a 50% or 60% antioxidant effect (EC_{50}) from all obtained films, 5 10, 20, 30, and 40 mg of granule film were placed in dark vials and three replicates were performed for each sample. Thereafter, 3 mL of [DPPH$^\bullet$] free radical methanolic solution and 2 mL of acetate buffer 100 mM (pH = 7.10) were added to each vial, and the absorbance of the reaction mixture was measured at 517 nm after 24 h. For a blank sample, we used a vial containing 3 mL of [DPPH$^\bullet$] free radical methanolic solution and 2 mL of acetate buffer without the addition of any granule film. The % inhibition of [DPPH$^\bullet$] was calculated using the following equation:

$$\%\ \text{scavenged DPPH}^\bullet\ \text{at steady state} = \frac{A_0^{517} - A_{\text{sample}}^{517}}{A_0^{517}} \times 100 \qquad \text{(S7)}$$

Table S1. Statistical analysis results of tensile properties

Multiple Comparisons							
Tukey HSD							
Dependent Variable			Mean Difference (I-J)	Std. Error	Sig.	95% Confidence Interval	
						Lower Bound	Upper Bound
E_Modulus	G/Gl	G/Gl/Eg&NZ10	304,5200*	79.86529	0.014	43.3429	565.6971
		G/Gl/Eg&NZ15	346,0033*	69.16537	0.001	119.8174	572.1893
		G/Gl/Eg&NZ5	32.7533	74.70714	1.000	-211.5554	277.0621

		G/Gl/NZ10	-667,5717*	74.70714	0.000	-911.8804	-423.2629
		G/Gl/NZ5	-792,4633*	69.16537	0.000	-1018.6493	-566.2774
		G/Gl25/Cit&NZ10	-324,7867*	71.43368	0.002	-558.3905	-91.1828
		G/Gl25/Cit&NZ5	-193.2267	71.43368	0.163	-426.8305	40.3772
	G/Gl/Eg&NZ10	G/Gl	-304,5200*	79.86529	0.014	-565.6971	-43.3429
		G/Gl/Eg&NZ15	41.4833	69.16537	0.999	-184.7026	267.6693
		G/Gl/Eg&NZ5	-271,7667*	74.70714	0.021	-516.0754	-27.4579
		G/Gl/NZ10	-972,0917*	74.70714	0.000	-1216.4004	-727.7829
		G/Gl/NZ5	-1096,9833*	69.16537	0.000	-1323.1693	-870.7974
		G/Gl25/Cit&NZ10	-629,3067*	71.43368	0.000	-862.9105	-395.7028
		G/Gl25/Cit&NZ5	-497,7467*	71.43368	0.000	-731.3505	-264.1428
	G/Gl/Eg&NZ15	G/Gl	-346,0033*	69.16537	0.001	-572.1893	-119.8174
		G/Gl/Eg&NZ10	-41.4833	69.16537	0.999	-267.6693	184.7026
		G/Gl/Eg&NZ5	-313,2500*	63.13905	0.001	-519.7286	-106.7714
		G/Gl/NZ10	-1013,5750*	63.13905	0.000	-1220.0536	-807.0964
		G/Gl/NZ5	-1138,4667*	56.47329	0.000	-1323.1467	-953.7866
		G/Gl25/Cit&NZ10	-670,7900*	59.22968	0.000	-864.4841	-477.0959
		G/Gl25/Cit&NZ5	-539,2300*	59.22968	0.000	-732.9241	-345.5359
	G/Gl/Eg&NZ5	G/Gl	-32.7533	74.70714	1.000	-277.0621	211.5554

		G/Gl/Eg&NZ10	271,7667*	74.70714	0.021	27.4579	516.0754
		G/Gl/Eg&NZ15	313,2500*	63.13905	0.001	106.7714	519.7286
		G/Gl/NZ10	-700,3250*	69.16537	0.000	-926.5110	-474.1390
		G/Gl/NZ5	-825,2167*	63.13905	0.000	-1031.6953	-618.7381
		G/Gl25/Cit&NZ10	-357,5400*	65.61603	0.000	-572.1188	-142.9612
		G/Gl25/Cit&NZ5	-225,9800*	65.61603	0.034	-440.5588	-11.4012
	G/Gl/NZ10	G/Gl	667,5717*	74.70714	0.000	423.2629	911.8804
		G/Gl/Eg&NZ10	972,0917*	74.70714	0.000	727.7829	1216.4004
		G/Gl/Eg&NZ15	1013,5750*	63.13905	0.000	807.0964	1220.0536
		G/Gl/Eg&NZ5	700,3250*	69.16537	0.000	474.1390	926.5110
		G/Gl/NZ5	-124.8917	63.13905	0.513	-331.3703	81.5869
		G/Gl25/Cit&NZ10	342,7850*	65.61603	0.000	128.2062	557.3638
		G/Gl25/Cit&NZ5	474,3450*	65.61603	0.000	259.7662	688.9238
	G/Gl/NZ5	G/Gl	792,4633*	69.16537	0.000	566.2774	1018.6493
		G/Gl/Eg&NZ10	1096,9833*	69.16537	0.000	870.7974	1323.1693
		G/Gl/Eg&NZ15	1138,4667*	56.47329	0.000	953.7866	1323.1467
		G/Gl/Eg&NZ5	825,2167*	63.13905	0.000	618.7381	1031.6953
		G/Gl/NZ10	124.8917	63.13905	0.513	-81.5869	331.3703
		G/Gl25/Cit&NZ10	467,6767*	59.22968	0.000	273.9826	661.3708

		G/Gl25/Cit&NZ5	599,2367*	59.22968	0.000	405.5426	792.9308
	G/Gl25/Cit&NZ10	G/Gl	324,7867*	71.43368	0.002	91.1828	558.3905
		G/Gl/Eg&NZ10	629,3067*	71.43368	0.000	395.7028	862.9105
		G/Gl/Eg&NZ15	670,7900*	59.22968	0.000	477.0959	864.4841
		G/Gl/Eg&NZ5	357,5400*	65.61603	0.000	142.9612	572.1188
		G/Gl/NZ10	-342,7850*	65.61603	0.000	-557.3638	-128.2062
		G/Gl/NZ5	-467,6767*	59.22968	0.000	-661.3708	-273.9826
		G/Gl25/Cit&NZ5	131.5600	61.86339	0.423	-70.7469	333.8669
	G/Gl25/Cit&NZ5	G/Gl	193.2267	71.43368	0.163	-40.3772	426.8305
		G/Gl/Eg&NZ10	497,7467*	71.43368	0.000	264.1428	731.3505
		G/Gl/Eg&NZ15	539,2300*	59.22968	0.000	345.5359	732.9241
		G/Gl/Eg&NZ5	225,9800*	65.61603	0.034	11.4012	440.5588
		G/Gl/NZ10	-474,3450*	65.61603	0.000	-688.9238	-259.7662
		G/Gl/NZ5	-599,2367*	59.22968	0.000	-792.9308	-405.5426
		G/Gl25/Cit&NZ10	-131.5600	61.86339	0.423	-333.8669	70.7469
Tensile_Strength	G/Gl	G/Gl/Eg&NZ10	8,7600*	2.54166	0.033	0.4482	17.0718
		G/Gl/Eg&NZ15	9,9717*	2.20114	0.002	2.7734	17.1699
		G/Gl/Eg&NZ5	1.5167	2.37751	0.998	-6.2583	9.2916
		G/Gl/NZ10	-16,4433*	2.37751	0.000	-24.2183	-8.6684
		G/Gl/NZ5	-23,5183*	2.20114	0.000	-30.7166	-16.3201

		G/Gl25/Cit&NZ10	-8,1933*	2.27333	0.023	-15.6276	-0.7590
		G/Gl25/Cit&NZ5	-7.3733	2.27333	0.053	-14.8076	0.0610
	G/Gl/Eg&NZ10	G/Gl	-8,7600*	2.54166	0.033	-17.0718	-0.4482
		G/Gl/Eg&NZ15	1.2117	2.20114	0.999	-5.9866	8.4099
		G/Gl/Eg&NZ5	-7.2433	2.37751	0.082	-15.0183	0.5316
		G/Gl/NZ10	-25,2033*	2.37751	0.000	-32.9783	-17.4284
		G/Gl/NZ5	-32,2783*	2.20114	0.000	-39.4766	-25.0801
		G/Gl25/Cit&NZ10	-16,9533*	2.27333	0.000	-24.3876	-9.5190
		G/Gl25/Cit&NZ5	-16,1333*	2.27333	0.000	-23.5676	-8.6990
	G/Gl/Eg&NZ15	G/Gl	-9,9717*	2.20114	0.002	-17.1699	-2.7734
		G/Gl/Eg&NZ10	-1.2117	2.20114	0.999	-8.4099	5.9866
		G/Gl/Eg&NZ5	-8,4550*	2.00936	0.005	-15.0260	-1.8840
		G/Gl/NZ10	-26,4150*	2.00936	0.000	-32.9860	-19.8440
		G/Gl/NZ5	-33,4900*	1.79723	0.000	-39.3673	-27.6127
		G/Gl25/Cit&NZ10	-18,1650*	1.88495	0.000	-24.3292	-12.0008
		G/Gl25/Cit&NZ5	-17,3450*	1.88495	0.000	-23.5092	-11.1808
	G/Gl/Eg&NZ5	G/Gl	-1.5167	2.37751	0.998	-9.2916	6.2583
		G/Gl/Eg&NZ10	7.2433	2.37751	0.082	-0.5316	15.0183
		G/Gl/Eg&NZ15	8,4550*	2.00936	0.005	1.8840	15.0260

		G/Gl/NZ10	-17,9600*	2.20114	0.000	-25.1582	-10.7618
		G/Gl/NZ5	-25,0350*	2.00936	0.000	-31.6060	-18.4640
		G/Gl25/Cit&NZ10	-9,7100*	2.08819	0.002	-16.5388	-2.8812
		G/Gl25/Cit&NZ5	-8,8900*	2.08819	0.005	-15.7188	-2.0612
	G/Gl/NZ10	G/Gl	16,4433*	2.37751	0.000	8.6684	24.2183
		G/Gl/Eg&NZ10	25,2033*	2.37751	0.000	17.4284	32.9783
		G/Gl/Eg&NZ15	26,4150*	2.00936	0.000	19.8440	32.9860
		G/Gl/Eg&NZ5	17,9600*	2.20114	0.000	10.7618	25.1582
		G/Gl/NZ5	-7,0750*	2.00936	0.028	-13.6460	-0.5040
		G/Gl25/Cit&NZ10	8,2500*	2.08819	0.010	1.4212	15.0788
		G/Gl25/Cit&NZ5	9,0700*	2.08819	0.004	2.2412	15.8988
	G/Gl/NZ5	G/Gl	23,5183*	2.20114	0.000	16.3201	30.7166
		G/Gl/Eg&NZ10	32,2783*	2.20114	0.000	25.0801	39.4766
		G/Gl/Eg&NZ15	33,4900*	1.79723	0.000	27.6127	39.3673
		G/Gl/Eg&NZ5	25,0350*	2.00936	0.000	18.4640	31.6060
		G/Gl/NZ10	7,0750*	2.00936	0.028	0.5040	13.6460
		G/Gl25/Cit&NZ10	15,3250*	1.88495	0.000	9.1608	21.4892
		G/Gl25/Cit&NZ5	16,1450*	1.88495	0.000	9.9808	22.3092
	G/Gl25/Cit&NZ10	G/Gl	8,1933*	2.27333	0.023	0.7590	15.6276

		G/Gl/Eg&NZ10	16,9533*	2.27333	0.000	9.5190	24.3876
		G/Gl/Eg&NZ15	18,1650*	1.88495	0.000	12.0008	24.3292
		G/Gl/Eg&NZ5	9,7100*	2.08819	0.002	2.8812	16.5388
		G/Gl/NZ10	-8,2500*	2.08819	0.010	-15.0788	-1.4212
		G/Gl/NZ5	-15,3250*	1.88495	0.000	-21.4892	-9.1608
		G/Gl25/Cit&NZ5	0.8200	1.96876	1.000	-5.6183	7.2583
	G/Gl25/Cit&NZ5	G/Gl	7.3733	2.27333	0.053	-0.0610	14.8076
		G/Gl/Eg&NZ10	16,1333*	2.27333	0.000	8.6990	23.5676
		G/Gl/Eg&NZ15	17,3450*	1.88495	0.000	11.1808	23.5092
		G/Gl/Eg&NZ5	8,8900*	2.08819	0.005	2.0612	15.7188
		G/Gl/NZ10	-9,0700*	2.08819	0.004	-15.8988	-2.2412
		G/Gl/NZ5	-16,1450*	1.88495	0.000	-22.3092	-9.9808
		G/Gl25/Cit&NZ10	-0.8200	1.96876	1.000	-7.2583	5.6183
Elongation	G/Gl	G/Gl/Eg&NZ10	-148,9567*	28.75791	0.000	-243.0013	-54.9120
		G/Gl/Eg&NZ15	-261,9900*	24.90508	0.000	-343.4351	-180.5449
		G/Gl/Eg&NZ5	-76.1067	26.90056	0.128	-164.0774	11.8641
		G/Gl/NZ10	61.6158	26.90056	0.333	-26.3549	149.5866
		G/Gl/NZ5	59.7800	24.90508	0.280	-21.6651	141.2251
		G/Gl25/Cit&NZ10	56.1033	25.72185	0.392	-28.0128	140.2195

		G/Gl25/Cit&NZ5	-45.2367	25.72185	0.651	-129.3528	38.8795
	G/Gl/Eg&NZ10	G/Gl	148,9567*	28.75791	0.000	54.9120	243.0013
		G/Gl/Eg&NZ15	-113,0333*	24.90508	0.002	-194.4784	-31.5883
		G/Gl/Eg&NZ5	72.8500	26.90056	0.162	-15.1207	160.8207
		G/Gl/NZ10	210,5725*	26.90056	0.000	122.6018	298.5432
		G/Gl/NZ5	208,7367*	24.90508	0.000	127.2916	290.1817
		G/Gl25/Cit&NZ10	205,0600*	25.72185	0.000	120.9439	289.1761
		G/Gl25/Cit&NZ5	103,7200*	25.72185	0.008	19.6039	187.8361
	G/Gl/Eg&NZ15	G/Gl	261,9900*	24.90508	0.000	180.5449	343.4351
		G/Gl/Eg&NZ10	113,0333*	24.90508	0.002	31.5883	194.4784
		G/Gl/Eg&NZ5	185,8833*	22.73512	0.000	111.5345	260.2322
		G/Gl/NZ10	323,6058*	22.73512	0.000	249.2570	397.9547
		G/Gl/NZ5	321,7700*	20.33491	0.000	255.2704	388.2696
		G/Gl25/Cit&NZ10	318,0933*	21.32743	0.000	248.3479	387.8387
		G/Gl25/Cit&NZ5	216,7533*	21.32743	0.000	147.0079	286.4987
	G/Gl/Eg&NZ5	G/Gl	76.1067	26.90056	0.128	-11.8641	164.0774
		G/Gl/Eg&NZ10	-72.8500	26.90056	0.162	-160.8207	15.1207
		G/Gl/Eg&NZ15	-185,8833*	22.73512	0.000	-260.2322	-111.5345
		G/Gl/NZ10	137,7225*	24.90508	0.000	56.2774	219.1676
		G/Gl/NZ5	135,8867*	22.73512	0.000	61.5378	210.2355

		G/Gl25/Cit&NZ10	132,2100*	23.62703	0.000	54.9444	209.4756
		G/Gl25/Cit&NZ5	30.8700	23.62703	0.889	-46.3956	108.1356
	G/Gl/NZ10	G/Gl	-61.6158	26.90056	0.333	-149.5866	26.3549
		G/Gl/Eg&NZ10	-210,5725*	26.90056	0.000	-298.5432	-122.6018
		G/Gl/Eg&NZ15	-323,6058*	22.73512	0.000	-397.9547	-249.2570
		G/Gl/Eg&NZ5	-137,7225*	24.90508	0.000	-219.1676	-56.2774
		G/Gl/NZ5	-1.8358	22.73512	1.000	-76.1847	72.5130
		G/Gl25/Cit&NZ10	-5.5125	23.62703	1.000	-82.7781	71.7531
		G/Gl25/Cit&NZ5	-106,8525*	23.62703	0.002	-184.1181	-29.5869
	G/Gl/NZ5	G/Gl	-59.7800	24.90508	0.280	-141.2251	21.6651
		G/Gl/Eg&NZ10	-208,7367*	24.90508	0.000	-290.1817	-127.2916
		G/Gl/Eg&NZ15	-321,7700*	20.33491	0.000	-388.2696	-255.2704
		G/Gl/Eg&NZ5	-135,8867*	22.73512	0.000	-210.2355	-61.5378
		G/Gl/NZ10	1.8358	22.73512	1.000	-72.5130	76.1847
		G/Gl25/Cit&NZ10	-3.6767	21.32743	1.000	-73.4221	66.0687
		G/Gl25/Cit&NZ5	-105,0167*	21.32743	0.001	-174.7621	-35.2713
	G/Gl25/Cit&NZ10	G/Gl	-56.1033	25.72185	0.392	-140.2195	28.0128
		G/Gl/Eg&NZ10	-205,0600*	25.72185	0.000	-289.1761	-120.9439
		G/Gl/Eg&NZ15	-318,0933*	21.32743	0.000	-387.8387	-248.3479

		G/Gl/Eg&NZ5	-132,2100*	23.62703	0.000	-209.4756	-54.9444
		G/Gl/NZ10	5.5125	23.62703	1.000	-71.7531	82.7781
		G/Gl/NZ5	3.6767	21.32743	1.000	-66.0687	73.4221
		G/Gl25/Cit&NZ5	-101,3400*	22.27578	0.002	-174.1867	-28.4933
	G/Gl25/Cit&NZ5	G/Gl	45.2367	25.72185	0.651	-38.8795	129.3528
		G/Gl/Eg&NZ10	-103,7200*	25.72185	0.008	-187.8361	-19.6039
		G/Gl/Eg&NZ15	-216,7533*	21.32743	0.000	-286.4987	-147.0079
		G/Gl/Eg&NZ5	-30.8700	23.62703	0.889	-108.1356	46.3956
		G/Gl/NZ10	106,8525*	23.62703	0.002	29.5869	184.1181
		G/Gl/NZ5	105,0167*	21.32743	0.001	35.2713	174.7621
		G/Gl25/Cit&NZ10	101,3400*	22.27578	0.002	28.4933	174.1867
Based on observed means.<br>The error term is Mean Square(Error) = 1240,526.							
*. The mean difference is significant at the ,05 level.							

Table S2. Experimental data used for the calculation of obtained average EC_{60} values

					% AA			
	**mg Δείγματος**	**%AA_1**	**%AA_2**	**%AA_3**	**average**	**stdev**	**EC60 mg/L**	
							**average**	**stdev**
**Gel/Gl/5EG@NZ**	4	51.53338	54.18974	53.65847	53.12719606	1.405613486	**7.491035335**	**0.853367**
	6	56.76511	59.69115	59.10594	58.52073085	1.548313004	8.400644345	
	8	59.8837	62.97048	62.35313	61.7357695	1.633374931	6.70806434	
	10	62.08204	65.28215	64.64213	64.00210822	1.693336617	7.364397321	
		1	2	3			**average**	**stdev**
**Gel/Gl/10EG@NZ**	4	53.0951	53.63689	56.88761	54.53986551	2.051171506	**9.606033974**	**3.091803**
	6	56.51388	55.97387	60.55058	57.67944284	2.501098104	10.10775246	
	8	57.64356	56.74609	61.76096	58.7168684	2.67417832	12.41629464	

	10	59.70969	58.15843	63.97467	60.61426513	3.011783898	6.294054824	
		1	2	3			**average**	**stdev**
**Gel/Gl/15EG@NZ**	4	50.35489	53.87704	49.69242	51.30811738	2.249278006	**9.927089559**	**2.39853**
	6	53.17811	59.04419	54.45823	55.56017674	3.084385577	12.0335695	
	8	54.91836	60.977	56.24092	57.37876046	3.185556716	7.316607077	
	10	57.6787	64.04767	59.07309	60.26648829	3.347996423	10.4310921	
		1	2	3			**average**	**stdev**
**Gel/Gl/5CT@NZ**	10	4.577402	4.272242	4.490214	4.446619217	0.157181506	**201.6003256**	**7.386796**
	20	6.819395	6.364769	6.689502	6.62455516	0.234168366	195.5391043	
	30	11.52135	10.75326	11.3019	11.19217082	0.395626919	209.8281418	
	40	12.98488	12.11922	12.73754	12.613879	0.445882231	199.4337308	
		1	2	3			**average**	**stdev**
**Gel/Gl/10CT@NZ**	10	5.035142	4.699466	4.939235	4.891281139	0.172899656	**182.8014952**	**6.747358**
	20	7.501335	7.001246	7.358452	7.287010676	0.257585202	177.2657959	
	30	12.67349	11.82859	12.43209	12.3113879	0.435189611	190.3173828	
	40	14.28336	13.33114	14.0113	13.8752669	0.490470454	180.8213069	

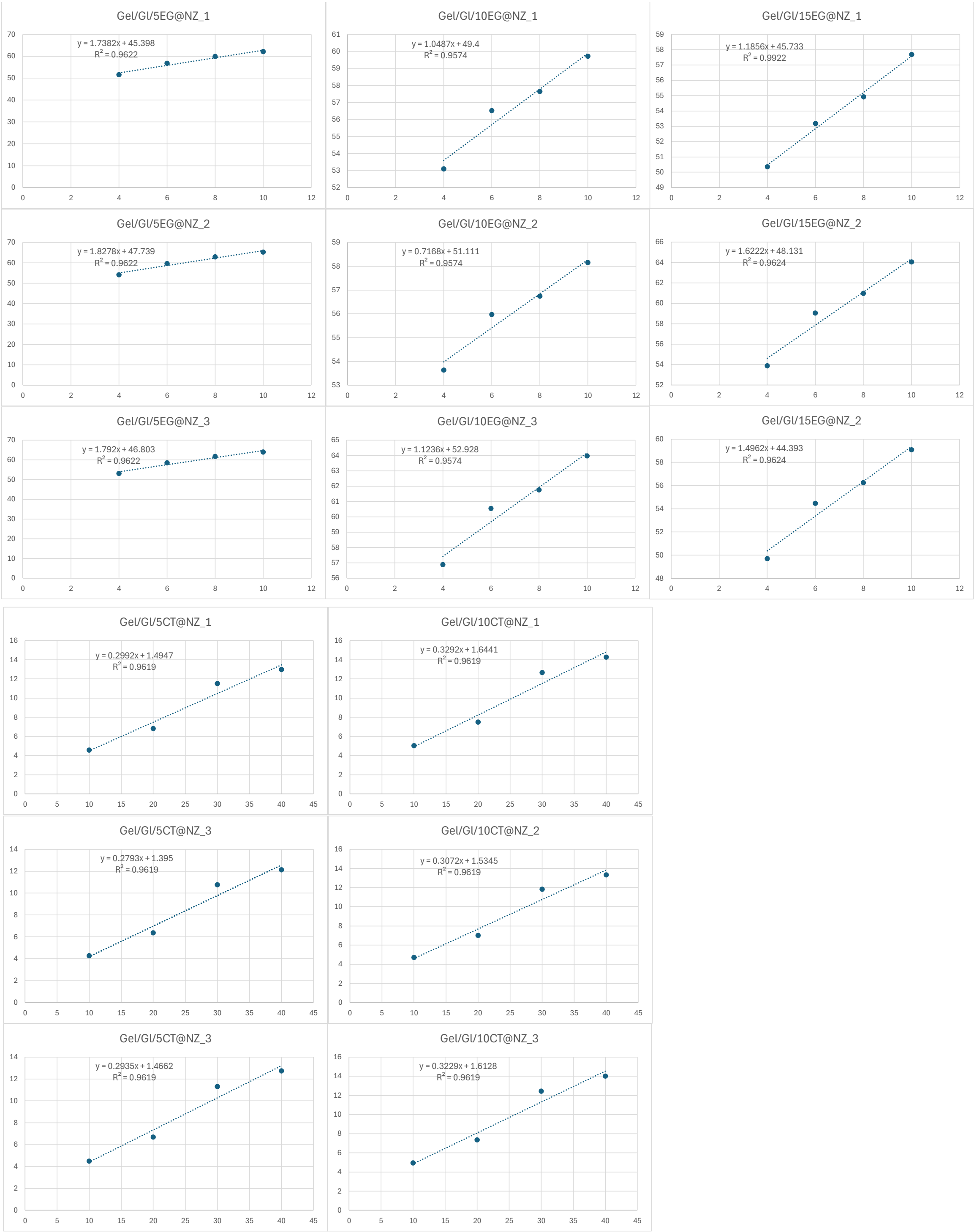

Figure S1. Linear plots used for the calculation of average values of EC_{60}.

Table S3: Statistical analysis results of EC_{60} values

Dependent Variable:						
Tukey HSD						
(I) Treatment		Mean Difference (I-J)	Std. Error	Sig.	95% Confidence Interval	
					Lower Bound	Upper Bound
Gel/Gl/10CT@NZ	Gel/Gl/10EG@NZ	-48,296373815896980*	0.782537533411778	0.000	-50.531372445589800	-46.061375186204200
	Gel/Gl/15EG@NZ	-46,537149064228690*	0.782537533411778	0.000	-48.772147693921500	-44.302150434535900
	Gel/Gl/5CT@NZ	0.871930604982206	0.782537533411778	0.798	-1.363068024710580	3.106929234674990
	Gel/Gl/5EG@NZ	-49,755214504717870*	0.782537533411778	0.000	-51.990213134410700	-47.520215875025100
Gel/Gl/10EG@NZ	Gel/Gl/10CT@NZ	48,296373815896980*	0.782537533411778	0.000	46.061375186204200	50.531372445589800
	Gel/Gl/15EG@NZ	1.759224751668280	0.782537533411778	0.183	-0.475773878024496	3.994223381361070
	Gel/Gl/5CT@NZ	49,168304420879180*	0.782537533411778	0.000	46.933305791186400	51.403303050572000
	Gel/Gl/5EG@NZ	-1.458840688820890	0.782537533411778	0.353	-3.693839318513670	0.776157940871888
Gel/Gl/15EG@NZ	Gel/Gl/10CT@NZ	46,537149064228690*	0.782537533411778	0.000	44.302150434535900	48.772147693921500
	Gel/Gl/10EG@NZ	-1.759224751668280	0.782537533411778	0.183	-3.994223381361070	0.475773878024496
	Gel/Gl/5CT@NZ	47,409079669210900*	0.782537533411778	0.000	45.174081039518100	49.644078298903700
	Gel/Gl/5EG@NZ	-3,218065440489177*	0.782537533411778	0.002	-5.453064070181960	-0.983066810796396

Gel/Gl/5CT@NZ	Gel/Gl/10CT@NZ	-0.871930604982206	0.782537533411778	0.798	-3.106929234674990	1.363068024710580
	Gel/Gl/10EG@NZ	-49,168304420879180*	0.782537533411778	0.000	-51.403303050572000	-46.933305791186400
	Gel/Gl/15EG@NZ	-47,409079669210900*	0.782537533411778	0.000	-49.644078298903700	-45.174081039518100
	Gel/Gl/5EG@NZ	-50,627145109700080*	0.782537533411778	0.000	-52.862143739392900	-48.392146480007300
Gel/Gl/5EG@NZ	Gel/Gl/10CT@NZ	49,755214504717870*	0.782537533411778	0.000	47.520215875025100	51.990213134410700
	Gel/Gl/10EG@NZ	1.458840688820890	0.782537533411778	0.353	-0.776157940871888	3.693839318513670
	Gel/Gl/15EG@NZ	3,218065440489177*	0.782537533411778	0.002	0.983066810796396	5.453064070181960
	Gel/Gl/5CT@NZ	50,627145109700080*	0.782537533411778	0.000	48.392146480007300	52.862143739392900
Based on observed means. The error term is Mean Square(Error) = 3,674.						
*. The mean difference is significant at the 0,05 level.						

**Multiple Comparisons**						
Dependent Variable:						
Tukey HSD						
(I) mg		Mean Difference (I-J)	Std. Error	Sig.	95% Confidence Interval	
					Lower Bound	Upper Bound
4mg	6mg	-4,261723827966541*	0.903596511132551	0.001	-7.065685775088210	-1.457761880844870
	8mg	-6,285406469228974*	0.903596511132551	0.000	-9.089368416350640	-3.481444522107300

	10mg	14,147573917861934*	0.808201289249612	0.000	11.639634109827200	16.655513725896600
	20mg	46,035943399752000*	1.010251611562020	0.000	42.901018639708600	49.170868159795400
	30mg	41,239946958470850*	1.010251611562020	0.000	38.105022198427500	44.374871718514200
	40mg	39,747153364164800*	1.010251611562020	0.000	36.612228604121400	42.882078124208200
6mg	4mg	4,261723827966542*	0.903596511132551	0.001	1.457761880844870	7.065685775088210
	8mg	##########	0.903596511132551	0.298	-4.827644588384100	0.780279305859239
	10mg	18,409297745828475*	0.808201289249612	0.000	15.901357937793800	20.917237553863200
	20mg	50,297667227718534*	1.010251611562020	0.000	47.162742467675200	53.432591987761900
	30mg	45,501670786437400*	1.010251611562020	0.000	42.366746026394000	48.636595546480800
	40mg	44,008877192131344*	1.010251611562020	0.000	40.873952432088000	47.143801952174700
8mg	4mg	6,285406469228974*	0.903596511132551	0.000	3.481444522107300	9.089368416350640
	6mg	##########	0.903596511132551	0.298	-0.780279305859239	4.827644588384100
	10mg	20,432980387090907*	0.808201289249612	0.000	17.925040579056200	22.940920195125600
	20mg	52,321349868980974*	1.010251611562020	0.000	49.186425108937600	55.456274629024300

	30mg	47,525353427699834*	1.010251611562020	0.000	44.390428667656500	50.660278187743200
	40mg	46,032559833393776*	1.010251611562020	0.000	42.897635073350400	49.167484593437100
10mg	4mg	-14,147573917861934*	0.808201289249612	0.000	-16.655513725896600	-11.639634109827200
	6mg	-18,409297745828475*	0.808201289249612	0.000	-20.917237553863200	-15.901357937793800
	8mg	-20,432980387090907*	0.808201289249612	0.000	-22.940920195125600	-17.925040579056200
	20mg	31,888369481890063*	0.925910896186812	0.000	29.015163479712100	34.761575484068100
	30mg	27,092373040608926*	0.925910896186812	0.000	24.219167038430900	29.965579042786900
	40mg	25,599579446302870*	0.925910896186812	0.000	22.726373444124900	28.472785448480900
20mg	4mg	-46,035943399752000*	1.010251611562020	0.000	-49.170868159795400	-42.901018639708600
	6mg	-50,297667227718534*	1.010251611562020	0.000	-53.432591987761900	-47.162742467675200
	8mg	-52,321349868980974*	1.010251611562020	0.000	-55.456274629024300	-49.186425108937600
	10mg	-31,888369481890063*	0.925910896186812	0.000	-34.761575484068100	-29.015163479712100
	30mg	-4,795996441281138*	1.106675192816920	0.002	-8.230134455595580	-1.361858426966700
	40mg	-6,288790035587196*	1.106675192816920	0.000	-9.722928049901640	-2.854652021272750
30mg	4mg	-41,239946958470850*	1.010251611562020	0.000	-44.374871718514200	-38.105022198427500
	6mg	-45,501670786437400*	1.010251611562020	0.000	-48.636595546480800	-42.366746026394000
	8mg	-47,525353427699834*	1.010251611562020	0.000	-50.660278187743200	-44.390428667656500
	10mg	-27,092373040608926*	0.925910896186812	0.000	-29.965579042786900	-24.219167038430900
	20mg	4,795996441281138*	1.106675192816920	0.002	1.361858426966700	8.230134455595580
	40mg	##########	1.106675192816920	0.825	-4.926931608620500	1.941344420008380
40mg	4mg	-39,747153364164800*	1.010251611562020	0.000	-42.882078124208200	-36.612228604121400

	6mg	-44,008877192131344*	1.010251611562020	0.000	-47.143801952174700	-40.873952432088000
	8mg	-46,032559833393776*	1.010251611562020	0.000	-49.167484593437100	-42.897635073350400
	10mg	-25,599579446302870*	0.925910896186812	0.000	-28.472785448480900	-22.726373444124900
	20mg	6,288790035587196*	1.106675192816920	0.000	2.854652021272750	9.722928049901640
	30mg	##########	1.106675192816920	0.825	-1.941344420008380	4.926931608620500
Based on observed means.<br>The error term is Mean Square(Error) = 3,674.						
*. The mean difference is significant at the 0,05 level.						

Antibacterial activity of films

Frozen stock cultures of *Escherichia coli* ATCC 25922 and *Listeria monocytogenes* WDCM 00021 were streaked onto Tryptone Soya Agar (TSA; Oxoid, UK) and incubated at 37 °C for 24 hours. Single colonies were transferred to Brain Heart Infusion Broth (BHI; Oxoid, UK) and incubated under the same conditions. Cultures were then centrifuged (6,500 × g, 10 min), resuspended in Davis Minimal Broth (DMB; Sigma, UK), and adjusted to OD_{600} = 1.0 before use.

The method was adapted from Turalija et al. (2016) and Ardjoum et al. (2021) *[11,12]*. Briefly, each film (0.25 g) was cut into small pieces and placed in individual sterile falcon tubes containing 5 mL of bacterial suspension (10^5 CFU/mL) in DMB. The tubes were incubated at 30°C with gentle agitation (120 rpm) for 18 hours. After incubation, bacterial suspensions were serially diluted in 0.8% saline and spread-plated on Nutrient Agar or TSA (Oxoid, UK). Plates were incubated at 37°C for 24 hours before enumeration, with microbial counts expressed as log CFU/mL using equation S1:

$$log\ CFU\ /\ mL\ = \frac{log10\ (number\ of\ colonies\ counted}{(dilution\ factor \times volume\ plated\ (mL))} \qquad (S8)$$

Table S4: Statistical analysis results of antibacterial activity results

Antibacterial activity of films against *S.* Typhimurium

Tukey's multiple comparisons test	Mean Diff.	95.00% CI of diff.	Below threshold?	Summary	Adjusted P Value
control vs. G/G125/NZ5	0.0333	-0.1491 to 0.2158	No	ns	0.997732
control vs. G/G125/NZ10	0.0667	-0.1158 to 0.2491	No	ns	0.899027
control vs. G/G125/10EG@NZ	5.4330	5.251 to 5.616	Yes	*	<0.000001
control vs. G/G125/15EG@NZ	5.4330	5.251 to 5.616	Yes	*	<0.000001
control vs. G/G125/5EG@NZ	5.4330	5.251 to 5.616	Yes	*	<0.000001
control vs. G/G125/10CT@NZ	0.0000	-0.1825 to 0.1825	No	ns	>0.999999
control vs. G/G125/5CT@NZ	0.0667	-0.1158 to 0.2491	No	ns	0.899027
G/G125/NZ5 vs. G/G125/NZ10	0.0333	-0.1491 to 0.2158	No	ns	0.997732
G/G125/NZ5 vs. G/G125/10EG@NZ	5.4000	5.218 to 5.582	Yes	*	<0.000001

G/G125/NZ5 vs. G/G125/15EG@NZ	5.4000	5.218 to 5.582	Yes	*	<0.000001
G/G125/NZ5 vs. G/G125/5EG@NZ	5.4000	5.218 to 5.582	Yes	*	<0.000001
G/G125/NZ5 vs. G/G125/10CT@NZ	-0.0333	-0.2158 to 0.1491	No	ns	0.997732
G/G125/NZ5 vs. G/G125/5CT@NZ	0.0333	-0.1491 to 0.2158	No	ns	0.997732
G/G125/NZ10 vs. G/G125/10EG@NZ	5.3670	5.184 to 5.549	Yes	*	<0.000001
G/G125/NZ10 vs. G/G125/15EG@NZ	5.3670	5.184 to 5.549	Yes	*	<0.000001
G/G125/NZ10 vs. G/G125/5EG@NZ	5.3670	5.184 to 5.549	Yes	*	<0.000001
G/G125/NZ10 vs. G/G125/10CT@NZ	-0.0667	-0.2491 to 0.1158	No	ns	0.899027
G/G125/NZ10 vs. G/G125/5CT@NZ	0.0000	-0.1825 to 0.1825	No	ns	>0.999999
G/G125/10EG@NZ vs. G/G125/15EG@NZ	0.0000	-0.1825 to 0.1825	No	ns	>0.999999
G/G125/10EG@NZ vs. G/G125/5EG@NZ	0.0000	-0.1825 to 0.1825	No	ns	>0.999999
G/G125/10EG@NZ vs. G/G125/10CT@NZ	-5.4330	-5.616 to -5.251	Yes	*	<0.000001
G/G125/10EG@NZ vs. G/G125/5CT@NZ	-5.3670	-5.549 to -5.184	Yes	*	<0.000001
G/G125/15EG@NZ vs. G/G125/5EG@NZ	0.0000	-0.1825 to 0.1825	No	ns	>0.999999
G/G125/15EG@NZ vs. G/G125/10CT@NZ	-5.4330	-5.616 to -5.251	Yes	*	<0.000001
G/G125/15EG@NZ vs. G/G125/5CT@NZ	-5.3670	-5.549 to -5.184	Yes	*	<0.000001
G/G125/5EG@NZ vs. G/G125/10CT@NZ	-5.4330	-5.616 to -5.251	Yes	*	<0.000001
G/G125/5EG@NZ vs. G/G125/5CT@NZ	-5.3670	-5.549 to -5.184	Yes	*	<0.000001
G/G125/10CT@NZ vs. G/G125/5CT@NZ	0.0667	-0.1158 to 0.2491	No	ns	0.899027

Antibacterial activity of films against *L. monocytogenes*

Tukey's multiple comparisons test	Mean Diff.	95.00% CI of diff.	Below threshold?	Summary	Adjusted P Value
control vs. G/G125/NZ5	0.0000	-0.3053 to 0.3053	No	ns	>0.999999
control vs. G/G125/NZ10	0.0000	-0.3053 to 0.3053	No	ns	>0.999999
control vs. G/G125/10EG@NZ	6.2330	5.928 to 6.539	Yes	*	<0.000001
control vs. G/G125/15EG@NZ	6.2330	5.928 to 6.539	Yes	*	<0.000001
control vs. G/G125/5EG@NZ	4.3670	4.061 to 4.672	Yes	*	<0.000001
control vs. G/G125/10CT@NZ	-0.0667	-0.3720 to 0.2387	No	ns	0.993294
control vs. G/G125/5CT@NZ	-0.0667	-0.3720 to 0.2387	No	ns	0.993294
G/G125/NZ5 vs. G/G125/NZ10	0.0000	-0.3053 to 0.3053	No	ns	>0.999999
G/G125/NZ5 vs. G/G125/10EG@NZ	6.2330	5.928 to 6.539	Yes	*	<0.000001
G/G125/NZ5 vs. G/G125/15EG@NZ	6.2330	5.928 to 6.539	Yes	*	<0.000001
G/G125/NZ5 vs. G/G125/5EG@NZ	4.3670	4.061 to 4.672	Yes	*	<0.000001
G/G125/NZ5 vs. G/G125/10CT@NZ	-0.0667	-0.3720 to 0.2387	No	ns	0.993294
G/G125/NZ5 vs. G/G125/5CT@NZ	-0.0667	-0.3720 to 0.2387	No	ns	0.993294
G/G125/NZ10 vs. G/G125/10EG@NZ	6.2330	5.928 to 6.539	Yes	*	<0.000001
G/G125/NZ10 vs. G/G125/15EG@NZ	6.2330	5.928 to 6.539	Yes	*	<0.000001
G/G125/NZ10 vs. G/G125/5EG@NZ	4.3670	4.061 to 4.672	Yes	*	<0.000001
G/G125/NZ10 vs. G/G125/10CT@NZ	-0.0667	-0.3720 to 0.2387	No	ns	0.993294
G/G125/NZ10 vs. G/G125/5CT@NZ	-0.0667	-0.3720 to 0.2387	No	ns	0.993294
G/G125/10EG@NZ vs. G/G125/15EG@NZ	0.0000	-0.3053 to 0.3053	No	ns	>0.999999

G/G125/10EG@NZ vs. G/G125/5EG@NZ	-1.8670	-2.172 to -1.561	Yes	*	<0.000001
G/G125/10EG@NZ vs. G/G125/10CT@NZ	-6.3000	-6.605 to -5.995	Yes	*	<0.000001
G/G125/10EG@NZ vs. G/G125/5CT@NZ	-6.3000	-6.605 to -5.995	Yes	*	<0.000001
G/G125/15EG@NZ vs. G/G125/5EG@NZ	-1.8670	-2.172 to -1.561	Yes	*	<0.000001
G/G125/15EG@NZ vs. G/G125/10CT@NZ	-6.3000	-6.605 to -5.995	Yes	*	<0.000001
G/G125/15EG@NZ vs. G/G125/5CT@NZ	-6.3000	-6.605 to -5.995	Yes	*	<0.000001
G/G125/5EG@NZ vs. G/G125/10CT@NZ	-4.4330	-4.739 to -4.128	Yes	*	<0.000001
G/G125/5EG@NZ vs. G/G125/5CT@NZ	-4.4330	-4.739 to -4.128	Yes	*	<0.000001
G/G125/10CT@NZ vs. G/G125/5CT@NZ	0.0000	-0.3053 to 0.3053	No	ns	>0.999999

Thiobarbituric Acid Reactive Substances of packaged fresh pork ham slices

Thiobarbituric acid-reactive Substances (TBARS) were determined using the SHIMADZU UV-1900 UV/VIS Spectrometer according to the procedure described in a recent paper [26] and following the concept reported in other previous publications [13,14]. In detail, In brief, 2 g of meat sample was placed in a vial along with 5 mL of 10% (w/v) trichloroacetic acid (TCA) solution. The mixture was then vortexed for 5 min, and then 5 mL 0.02 M aqueous solution of 2-thiobarbituric acid was added to it and vortexed again for 5 min. The mixture was left in the dark for 18–24 h for color development. The resultant supernatant was pipetted off and centrifuged, and the absorbance (D) was measured against the blank sample, 5 mL of 10% (w/v) TCA solution, and 5 mL of 0.02 M aqueous solution, at λ = 538 nm using glass cuvettes of 1 cm. TBARS measurements were based on the adsorption Abs_{538} at λ = 538 nm and were expressed as mg of malondialdehyde per 1 kg of sample (mg_{MDA}/kg_{sample}) according to the following equation:

$$TBARS\ (mg_{MDA}/kg_{sample}) = 7.8 \times Abs_{538} \quad (S9)$$

where 7.8 is a calibration constant to transform the absorbance Abs_{538} to mg_{MDA}/kg_{sample} [14].

Table S5. Calculated TBA mean values of fresh pork ham slices with Gel/Gl/15EG@NZ, Gel/Gl/10CT@NZ active films applied as extra active pads as well as fresh pork ham slices without extra active pads (control sample) during the twenty-six days of storage at 4±1 °C

	TBA		
**Day of storage**	**Control**	**Gel/Gl/10CT@NZ**	**Gel/Gl/15EG@NZ**
**0**	0.068±0.017^{Ab}	0.076±0.006^{Bb}	0.077±0.006^{Bb}
**2**	0.050±0.027^{Ab}	0.066±0.005^{Bb}	0.034±0.005^{Cb}
**4**	0.072±0.018^{Aa}	0.144±0.059^{Ba}	0.154±0.006^{Ba}
**8**	0.045±0.017^{Ab}	0.072±0.01^{Bb}	0.032±0.010^{Cb}
**10**	0.031±0.020^{Ab}	0.088±0.027^{Bb}	0.075±0.028^{Cb}
**14**	0.038±0.019^{Ab}	0.060±0.003^{Cb}	0.043±0.003^{Cb}
**18**	0.034±0.019^{Ab}	0.043±0.002^{Bb}	0.066±0.002^{Cb}
**22**	0.059±0.017^{Ab}	0.061±0.003^{Bb}	0.060±0.003^{Cb}
**26**	0.042±0.021^{Ab}	0.063±0.003^{Bb}	0.054±0.003^{Cb}

Different capital letters in the same column indicate significant differences between treatments (Tukey HSD, p < 0.05). Different lowercase letters in the same row indicate significant differences between storage days for the same treatment.

Table S6: Statistical analysis results of TBA results

**Multiple Comparisons**						
Dependent Variable:						
Tukey HSD						
(I) Treatment		Mean Difference (I-J)	Std. Error	Sig.	95% Confidence Interval	
					Lower Bound	Upper Bound
Control	CT@NZ10	-,015692222222222*	0.004994542779450	0.006	-0.027576606867552	-0.003807837576893
	EG@NZ15	-,024581111111111*	0.004994542779450	0.000	-0.036465495756441	-0.012696726465782
CT@NZ10	Control	,015692222222222*	0.004994542779450	0.006	0.003807837576893	0.027576606867552
	EG@NZ15	-0.008888888888889	0.004708900091034	0.148	-0.020093594186145	0.002315816408367
EG@NZ15	Control	,024581111111111*	0.004994542779450	0.000	0.012696726465782	0.036465495756441
	CT@NZ10	0.008888888888889	0.004708900091034	0.148	-0.002315816408367	0.020093594186145
Based on observed means.<br>The error term is Mean Square(Error) = ,000.						
*. The mean difference is significant at the 0,05 level.						

**Multiple Comparisons**						
Dependent Variable:						
Tukey HSD						
(I) Day		Mean Difference (I-J)	Std. Error	Sig.	95% Confidence Interval	
					Lower Bound	Upper Bound
0	2	0.023771428571429	0.008442318160952	0.124	-0.003003446337142	0.050546303479999
	4	-,053228571428571*	0.008442318160952	0.000	-0.080003446337142	-0.026453696520001
	8	0.024271428571429	0.008442318160952	0.108	-0.002503446337142	0.051046303479999
	10	0.006414285714286	0.008442318160952	0.998	-0.020360589194285	0.033189160622857
	14	0.025700000000000	0.008442318160952	0.070	-0.001074874908571	0.052474874908571

	18	0.025271428571429	0.008442318160952	0.080	-0.001503446337142	0.052046303479999
	22	0.014128571428571	0.008442318160952	0.761	-0.012646303479999	0.040903446337142
	26	0.019771428571429	0.008442318160952	0.328	-0.007003446337142	0.046546303479999
2	0	-0.023771428571429	0.008442318160952	0.124	-0.050546303479999	0.003003446337142
	4	-,077000000000000*	0.008442318160952	0.000	-0.103774874908571	-0.050225125091429
	8	0.000500000000000	0.008442318160952	1.000	-0.026274874908571	0.027274874908571
	10	-0.017357142857143	0.008442318160952	0.509	-0.044132017765714	0.009417732051428
	14	0.001928571428571	0.008442318160952	1.000	-0.024846303479999	0.028703446337142
	18	0.001500000000000	0.008442318160952	1.000	-0.025274874908571	0.028274874908571
	22	-0.009642857142857	0.008442318160952	0.966	-0.036417732051428	0.017132017765714
	26	-0.004000000000000	0.008442318160952	1.000	-0.030774874908571	0.022774874908571
4	0	,053228571428571*	0.008442318160952	0.000	0.026453696520001	0.080003446337142
	2	,077000000000000*	0.008442318160952	0.000	0.050225125091429	0.103774874908571
	8	,077500000000000*	0.008442318160952	0.000	0.050725125091429	0.104274874908571
	10	,059642857142857*	0.008442318160952	0.000	0.032867982234286	0.086417732051428
	14	,078928571428571*	0.008442318160952	0.000	0.052153696520001	0.105703446337142
	18	,078500000000000*	0.008442318160952	0.000	0.051725125091429	0.105274874908571
	22	,067357142857143*	0.008442318160952	0.000	0.040582267948572	0.094132017765714
	26	,073000000000000*	0.008442318160952	0.000	0.046225125091429	0.099774874908571
8	0	-0.024271428571429	0.008442318160952	0.108	-0.051046303479999	0.002503446337142
	2	-0.000500000000000	0.008442318160952	1.000	-0.027274874908571	0.026274874908571
	4	-,077500000000000*	0.008442318160952	0.000	-0.104274874908571	-0.050725125091429
	10	-0.017857142857143	0.008442318160952	0.469	-0.044632017765714	0.008917732051428
	14	0.001428571428571	0.008442318160952	1.000	-0.025346303479999	0.028203446337142
	18	0.001000000000000	0.008442318160952	1.000	-0.025774874908571	0.027774874908571
	22	-0.010142857142857	0.008442318160952	0.954	-0.036917732051428	0.016632017765714
	26	-0.004500000000000	0.008442318160952	1.000	-0.031274874908571	0.022274874908571
10	0	-0.006414285714286	0.008442318160952	0.998	-0.033189160622857	0.020360589194285
	2	0.017357142857143	0.008442318160952	0.509	-0.009417732051428	0.044132017765714
	4	-,059642857142857*	0.008442318160952	0.000	-0.086417732051428	-0.032867982234286
	8	0.017857142857143	0.008442318160952	0.469	-0.008917732051428	0.044632017765714
	14	0.019285714285714	0.008442318160952	0.362	-0.007489160622857	0.046060589194285
	18	0.018857142857143	0.008442318160952	0.393	-0.007917732051428	0.045632017765714
	22	0.007714285714286	0.008442318160952	0.992	-0.019060589194285	0.034489160622857
	26	0.013357142857143	0.008442318160952	0.812	-0.013417732051428	0.040132017765714
14	0	-0.025700000000000	0.008442318160952	0.070	-0.052474874908571	0.001074874908571
	2	-0.001928571428571	0.008442318160952	1.000	-0.028703446337142	0.024846303479999
	4	-,078928571428571*	0.008442318160952	0.000	-0.105703446337142	-0.052153696520001
	8	-0.001428571428571	0.008442318160952	1.000	-0.028203446337142	0.025346303479999

	10	-0.019285714285714	0.008442318160952	0.362	-0.046060589194285	0.007489160622857
	18	-0.000428571428571	0.008442318160952	1.000	-0.027203446337142	0.026346303479999
	22	-0.011571428571429	0.008442318160952	0.906	-0.038346303479999	0.015203446337142
	26	-0.005928571428571	0.008442318160952	0.999	-0.032703446337142	0.020846303479999
18	0	-0.025271428571429	0.008442318160952	0.080	-0.052046303479999	0.001503446337142
	2	-0.001500000000000	0.008442318160952	1.000	-0.028274874908571	0.025274874908571
	4	-,078500000000000*	0.008442318160952	0.000	-0.105274874908571	-0.051725125091429
	8	-0.001000000000000	0.008442318160952	1.000	-0.027774874908571	0.025774874908571
	10	-0.018857142857143	0.008442318160952	0.393	-0.045632017765714	0.007917732051428
	14	0.000428571428571	0.008442318160952	1.000	-0.026346303479999	0.027203446337142
	22	-0.011142857142857	0.008442318160952	0.923	-0.037917732051428	0.015632017765714
	26	-0.005500000000000	0.008442318160952	0.999	-0.032274874908571	0.021274874908571
22	0	-0.014128571428571	0.008442318160952	0.761	-0.040903446337142	0.012646303479999
	2	0.009642857142857	0.008442318160952	0.966	-0.017132017765714	0.036417732051428
	4	-,067357142857143*	0.008442318160952	0.000	-0.094132017765714	-0.040582267948572
	8	0.010142857142857	0.008442318160952	0.954	-0.016632017765714	0.036917732051428
	10	-0.007714285714286	0.008442318160952	0.992	-0.034489160622857	0.019060589194285
	14	0.011571428571429	0.008442318160952	0.906	-0.015203446337142	0.038346303479999
	18	0.011142857142857	0.008442318160952	0.923	-0.015632017765714	0.037917732051428
	26	0.005642857142857	0.008442318160952	0.999	-0.021132017765714	0.032417732051428
26	0	-0.019771428571429	0.008442318160952	0.328	-0.046546303479999	0.007003446337142
	2	0.004000000000000	0.008442318160952	1.000	-0.022774874908571	0.030774874908571
	4	-,073000000000000*	0.008442318160952	0.000	-0.099774874908571	-0.046225125091429
	8	0.004500000000000	0.008442318160952	1.000	-0.022274874908571	0.031274874908571
	10	-0.013357142857143	0.008442318160952	0.812	-0.040132017765714	0.013417732051428
	14	0.005928571428571	0.008442318160952	0.999	-0.020846303479999	0.032703446337142
	18	0.005500000000000	0.008442318160952	0.999	-0.021274874908571	0.032274874908571
	22	-0.005642857142857	0.008442318160952	0.999	-0.032417732051428	0.021132017765714
Based on observed means. The error term is Mean Square(Error) = ,000.						
*. The mean difference is significant at the 0,05 level.						

Total Viable Count (TVC) of Pork Ham Slices

TVC was monitored with respect to storage time at 4 ± 1 °C (0, 2, 4, 8, 10, 14, 18, 22, and 26 days). Ten grams of pork ham slices were removed aseptically from each packaging system and transferred to a stomacher bag (Seward Medical, Worthing, West Sussex, UK), containing 90 mL of sterile buffered peptone water (BPW, NCM0015A, Heywood, BL97JJ, UK; 0.1 g/100 mL of distilled water) and homogenized using a stomacher (LAB Blender 400, Seward Medical, UK) for 90 s at room temperature. For the microbial enumeration, 0.1 mL of serial dilutions (1:10 diluents, buffered peptone water) of pork meat homogenates were spread on the surface of plate count agar (PCA, NCM0010A, Heywood UK). TVC was determined after incubation for 2 days at 30 °C [15].

Table S7: Statistical analysis results of TVC

**Multiple Comparisons**						
Dependent Variable:						
Tukey HSD						
(I) Day		Mean Difference (I-J)	Std. Error	Sig.	95% Confidence Interval	
					Lower Bound	Upper Bound
Day 0	Day 2	-,6511*	0.12688	0.000	-1.0610	-0.2412
	Day 4	-,9700*	0.12688	0.000	-1.3799	-0.5601
	Day 6	-1,7722*	0.12688	0.000	-2.1822	-1.3623
	Day 10	-1,9033*	0.12688	0.000	-2.3133	-1.4934
	14	-1,8689*	0.12688	0.000	-2.2788	-1.4590
	18	-2,6700*	0.12688	0.000	-3.0799	-2.2601
	22	-3,2967*	0.12688	0.000	-3.7066	-2.8867
	26	-4,2856*	0.12688	0.000	-4.6955	-3.8756
Day 2	Day 0	,6511*	0.12688	0.000	0.2412	1.0610
	Day 4	-0.3189	0.12688	0.249	-0.7288	0.0910
	Day 6	-1,1211*	0.12688	0.000	-1.5310	-0.7112
	Day 10	-1,2522*	0.12688	0.000	-1.6622	-0.8423
	14	-1,2178*	0.12688	0.000	-1.6277	-0.8078
	18	-2,0189*	0.12688	0.000	-2.4288	-1.6090
	22	-2,6456*	0.12688	0.000	-3.0555	-2.2356
	26	-3,6344*	0.12688	0.000	-4.0444	-3.2245
Day 4	Day 0	,9700*	0.12688	0.000	0.5601	1.3799
	Day 2	0.3189	0.12688	0.249	-0.0910	0.7288
	Day 6	-,8022*	0.12688	0.000	-1.2122	-0.3923
	Day 10	-,9333*	0.12688	0.000	-1.3433	-0.5234
	14	-,8989*	0.12688	0.000	-1.3088	-0.4890
	18	-1,7000*	0.12688	0.000	-2.1099	-1.2901
	22	-2,3267*	0.12688	0.000	-2.7366	-1.9167
	26	-3,3156*	0.12688	0.000	-3.7255	-2.9056
Day 6	Day 0	1,7722*	0.12688	0.000	1.3623	2.1822
	Day 2	1,1211*	0.12688	0.000	0.7112	1.5310

	Day 4	,8022*	0.12688	0.000	0.3923	1.2122
	Day 10	-0.1311	0.12688	0.981	-0.5410	0.2788
	14	-0.0967	0.12688	0.997	-0.5066	0.3133
	18	-,8978*	0.12688	0.000	-1.3077	-0.4878
	22	-1,5244*	0.12688	0.000	-1.9344	-1.1145
	26	-2,5133*	0.12688	0.000	-2.9233	-2.1034
Day 10	Day 0	1,9033*	0.12688	0.000	1.4934	2.3133
	Day 2	1,2522*	0.12688	0.000	0.8423	1.6622
	Day 4	,9333*	0.12688	0.000	0.5234	1.3433
	Day 6	0.1311	0.12688	0.981	-0.2788	0.5410
	14	0.0344	0.12688	1.000	-0.3755	0.4444
	18	-,7667*	0.12688	0.000	-1.1766	-0.3567
	22	-1,3933*	0.12688	0.000	-1.8033	-0.9834
	26	-2,3822*	0.12688	0.000	-2.7922	-1.9723
14	Day 0	1,8689*	0.12688	0.000	1.4590	2.2788
	Day 2	1,2178*	0.12688	0.000	0.8078	1.6277
	Day 4	,8989*	0.12688	0.000	0.4890	1.3088
	Day 6	0.0967	0.12688	0.997	-0.3133	0.5066
	Day 10	-0.0344	0.12688	1.000	-0.4444	0.3755
	18	-,8011*	0.12688	0.000	-1.2110	-0.3912
	22	-1,4278*	0.12688	0.000	-1.8377	-1.0178
	26	-2,4167*	0.12688	0.000	-2.8266	-2.0067
18	Day 0	2,6700*	0.12688	0.000	2.2601	3.0799
	Day 2	2,0189*	0.12688	0.000	1.6090	2.4288
	Day 4	1,7000*	0.12688	0.000	1.2901	2.1099
	Day 6	,8978*	0.12688	0.000	0.4878	1.3077
	Day 10	,7667*	0.12688	0.000	0.3567	1.1766
	14	,8011*	0.12688	0.000	0.3912	1.2110
	22	-,6267*	0.12688	0.000	-1.0366	-0.2167
	26	-1,6156*	0.12688	0.000	-2.0255	-1.2056
22	Day 0	3,2967*	0.12688	0.000	2.8867	3.7066
	Day 2	2,6456*	0.12688	0.000	2.2356	3.0555
	Day 4	2,3267*	0.12688	0.000	1.9167	2.7366
	Day 6	1,5244*	0.12688	0.000	1.1145	1.9344
	Day 10	1,3933*	0.12688	0.000	0.9834	1.8033
	14	1,4278*	0.12688	0.000	1.0178	1.8377

	18	,6267*	0.12688	0.000	0.2167	1.0366
	26	-,9889*	0.12688	0.000	-1.3988	-0.5790
26	Day 0	4,2856*	0.12688	0.000	3.8756	4.6955
	Day 2	3,6344*	0.12688	0.000	3.2245	4.0444
	Day 4	3,3156*	0.12688	0.000	2.9056	3.7255
	Day 6	2,5133*	0.12688	0.000	2.1034	2.9233
	Day 10	2,3822*	0.12688	0.000	1.9723	2.7922
	14	2,4167*	0.12688	0.000	2.0067	2.8266
	18	1,6156*	0.12688	0.000	1.2056	2.0255
	22	,9889*	0.12688	0.000	0.5790	1.3988
Based on observed means.<br>The error term is Mean Square(Error) = ,072.						
*. The mean difference is significant at the 0,05 level.						

Multiple Comparisons

Dependent Variable:

Tukey HSD

(I) Treatment		Mean Difference (I-J)	Std. Error	Sig.	95% Confidence Interval	
					Lower Bound	Upper Bound
Control	G_Gl_CT&NZ10	,7170*	0.07326	0.000	0.5405	0.8936
	G_Gl_EG&NZ15	2,2315*	0.07326	0.000	2.0549	2.4080
G_Gl_CT&NZ10	Control	-,7170*	0.07326	0.000	-0.8936	-0.5405
	G_Gl_EG&NZ15	1,5144*	0.07326	0.000	1.3379	1.6910
G_Gl_EG&NZ15	Control	-2,2315*	0.07326	0.000	-2.4080	-2.0549
	G_Gl_CT&NZ10	-1,5144*	0.07326	0.000	-1.6910	-1.3379
Based on observed means.<br>The error term is Mean Square(Error) = ,072.						
*. The mean difference is significant at the 0,05 level.						

References

1. Saleh, T.A. Chapter 3 - Kinetic Models and Thermodynamics of Adsorption Processes: Classification. In *Interface Science and Technology*; Saleh, T.A., Ed.; Surface Science of Adsorbents and Nanoadsorbents; Elsevier, 2022; Vol. 34, pp. 65–97.
2. Asimakopoulos, G.; Baikousi, M.; Salmas, C.; Bourlinos, A.B.; Zboril, R.; Karakassides, M.A. Advanced Cr(VI) Sorption Properties of Activated Carbon Produced via Pyrolysis of the “<i>Posidonia Oceanica”</i> Seagrass. *J. Hazard. Mater.* **2021**, *405*, 124274, doi:10.1016/j.jhazmat.2020.124274.
3. Frenkel, J. Theorie der Adsorption und verwandter Erscheinungen. *Z. Für Phys.* **1924**, *26*, 117–138, doi:10.1007/BF01327320.
4. Knopf, D.A.; Ammann, M. Technical Note: Adsorption and Desorption Equilibria from Statistical Thermodynamics and Rates from Transition State Theory. *Atmospheric Chem. Phys.* **2021**, *21*, 15725–15753, doi:10.5194/acp-21-15725-2021.
5. Arrhenius, S. Über die Dissociationswärme und den Einfluss der Temperatur auf den Dissociationsgrad der Elektrolyte. *Z. Für Phys. Chem.* **1889**, *4U*, 96–116, doi:10.1515/zpch-1889-0408.
6. Kechagias, A.; Salmas, C.E.; Chalmpes, N.; Leontiou, A.A.; Karakassides, M.A.; Giannelis, E.P.; Giannakas, A.E. Laponite vs. Montmorillonite as Eugenol Nanocarriers for Low Density Polyethylene Active Packaging Films. *Nanomaterials* **2024**, *14*, 1938, doi:10.3390/nano14231938.
7. Salmas, C.E.; Giannakas, A.E.; Baikousi, M.; Kollia, E.; Tsigkou, V.; Proestos, C. Effect of Copper and Titanium-Exchanged Montmorillonite Nanostructures on the Packaging Performance of Chitosan/Poly-Vinyl-Alcohol-Based Active Packaging Nanocomposite Films. *Foods* **2021**, *10*, 3038, doi:10.3390/foods10123038.
8. Karabagias, I.K.; Karabagias, V.K.; Badeka, A.V. In Search of the EC60: The Case Study of Bee Pollen, Quercus Ilex Honey, and Saffron. *Eur. Food Res. Technol.* **2020**, *246*, 2451–2459, doi:10.1007/s00217-020-03588-8.
9. Connolly, J.M.; Kane, M.T.; Quinlan, L.R.; Hynes, A.C. Enhancing Oxygen Delivery to Ovarian Follicles by Three Different Methods Markedly Improves Growth in Serum-Containing Culture Medium. *Reprod. Fertil. Dev.* **2019**, *31*, 1339–1352, doi:10.1071/RD18286.
10. Stratakos, A.C.; Koidis, A. Chapter 4 - Methods for Extracting Essential Oils. In *Essential Oils in Food Preservation, Flavor and Safety*; Preedy, V.R., Ed.; Academic Press: San Diego, 2016; pp. 31–38 ISBN 978-0-12-416641-7.
11. Turalija, M.; Bischof, S.; Budimir, A.; Gaan, S. Antimicrobial PLA Films from Environment Friendly Additives. *Compos. Part B Eng.* **2016**, *102*, 94–99, doi:10.1016/j.compositesb.2016.07.017.
12. Ardjoum, N.; Chibani, N.; Shankar, S.; Fadhel, Y.B.; Djidjelli, H.; Lacroix, M. Development of Antimicrobial Films Based on Poly(Lactic Acid) Incorporated with *Thymus Vulgaris* Essential Oil and Ethanolic Extract of Mediterranean Propolis. *Int. J. Biol. Macromol.* **2021**, *185*, 535–542, doi:10.1016/j.ijbiomac.2021.06.194.
13. Tarladgis, B.G.; Watts, B.M.; Younathan, M.T.; Dugan, L. A Distillation Method for the Quantitative Determination of Malonaldehyde in Rancid Foods. *J. Am. Oil Chem. Soc.* **1960**, *37*, 44–48, doi:10.1007/BF02630824.
14. Karabagias, I.; Badeka, A.; Kontominas, M.G. Shelf Life Extension of Lamb Meat Using Thyme or Oregano Essential Oils and Modified Atmosphere Packaging. *Meat Sci.* **2011**, *88*, 109–116, doi:10.1016/j.meatsci.2010.12.010.
15. Assanti, E.; Karabagias, V.K.; Karabagias, I.K.; Badeka, A.; Kontominas, M.G. Shelf Life Evaluation of Fresh Chicken Burgers Based on the Combination of Chitosan Dip and Vacuum Packaging under Refrigerated Storage. *J. Food Sci. Technol.* **2021**, *58*, 870–883, doi:10.1007/s13197-020-04601-4.
